# Selective hydrogenation of 1,3-butadiene on platinum–copper alloys at the single-atom limit

**DOI:** 10.1038/ncomms9550

**Published:** 2015-10-09

**Authors:** Felicia R. Lucci, Jilei Liu, Matthew D. Marcinkowski, Ming Yang, Lawrence F. Allard, Maria Flytzani-Stephanopoulos, E. Charles H. Sykes

**Affiliations:** 1 Department of Chemistry, Tufts University, 62 Talbot Avenue, Medford, Massachusetts 02155, USA; 2 Department of Chemical and Biological Engineering, Tufts University, 4 Colby Street, Medford, Massachusetts 02155, USA; 3 Materials Science and Technology Division, Oak Ridge National Laboratory, PO Box 2008 MS-6064, Oak Ridge, Tennessee 37831, USA

## Abstract

Platinum is ubiquitous in the production sectors of chemicals and fuels; however, its scarcity in nature and high price will limit future proliferation of platinum-catalysed reactions. One promising approach to conserve platinum involves understanding the smallest number of platinum atoms needed to catalyse a reaction, then designing catalysts with the minimal platinum ensembles. Here we design and test a new generation of platinum–copper nanoparticle catalysts for the selective hydrogenation of 1,3-butadiene,, an industrially important reaction. Isolated platinum atom geometries enable hydrogen activation and spillover but are incapable of C–C bond scission that leads to loss of selectivity and catalyst deactivation. γ-Alumina-supported single-atom alloy nanoparticle catalysts with <1 platinum atom per 100 copper atoms are found to exhibit high activity and selectivity for butadiene hydrogenation to butenes under mild conditions, demonstrating transferability from the model study to the catalytic reaction under practical conditions.

## Abstract (web summary)

Reducing the platinum content of industrial catalysts is an important research target. Here, the authors present a nanocatalyst containing less than 1% platinum, where the isolated platinum atoms contribute to both the catalyst activity and selectivity for butadiene hydrogenation.

## Graphical abstract

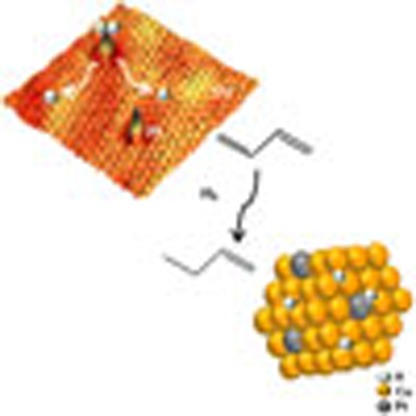

Platinum (Pt) is one of the most widely used transition metal catalysts due to its superior catalytic performance for both oxidation and hydrogenation reactions. It is used extensively in electrochemical and heterogeneous catalysts with applications in fuel cells, automotive catalytic converters and industrial hydrocarbon cracking processes^1^,^2^. Pt will also undoubtedly play a large role in the ongoing development of clean energy technologies. Despite its favourable reactivity, there are two major drawbacks of Pt catalysts. First, Pt is very expensive and scarce in nature, which imposes major limitations on its future role in catalysis. Second, Pt is susceptible to carbon monoxide (CO) poisoning in polymer electrolyte membrane (PEM) fuel cells because CO binds strongly to metallic Pt^3^,^4^. Therefore, a key step towards the next-generation catalysts will be the development of hybrid systems that retain the high activity of Pt while improving its selectivity and increasing its tolerance to CO poisoning.

One approach is to design catalysts that contain only the Pt sites necessary to perform or to assist a target reaction. Recently ‘single-site heterogeneous catalysts' containing atomically dispersed active metal species have been shown to be effective in catalysing a number of reactions^5^. Single-site Pt and Au cations stabilized by -O- linkages on various supports with vicinal -OH groups have been identified as the active sites for the water–gas shift reaction^6^,^7^. In a similar fashion, isolated precious metal-O_*x*_ species on various support matrices have shown superior activity for CO oxidation^8^, and the steam reforming of methanol^9^,^10^. Atomically dispersed Au in metallic Ni has been shown to markedly suppress carbon deposition in the steam reforming of methane^11^, whereas isolated Pd atoms in a metallic copper host catalyse selective hydrogenation reactions^12^,^13^,^14^. Although Pt and Pd are often interchangeable in terms of catalytic activity, they have different physical properties that impact their alloying behaviour, surface segregation and electronic structure that can potentially impact their ability to activate H_2_ and catalyse hydrogenation reactions^15^,^16^,^17^,^18^,^19^. Pt/Cu bimetallic alloys offer an alternative method for reducing the amount of Pt and potentially enhancing catalyst selectivity for partial hydrogenation reactions^20^,^21^,^22^,^23^, but to date it has not been reported that single metallic Pt atoms are capable of such chemistry. Theoretical work has shown that by simultaneously optimizing the activation of reactants and binding strength of intermediates, the catalyst activity for hydrogenation reactions should be controllable^24^.

Herein we show that very low concentrations of individual, isolated Pt atoms in a Cu surface catalyse the industrially important butadiene hydrogenation reaction with high selectivity to butenes. Butadiene poisons polymerization catalysts even at low concentrations (<10 p.p.m.) in industrial alkene streams^25^. Of particular interest is the butadiene impurity in propene feedstocks used to produce 42.3 million tons of polypropylene annually^26^. The selective hydrogenation of butadiene to butene serves to increase the purity of alkene feedstocks without reducing their overall concentration. Therefore, catalysts that selectively hydrogenate butadiene to butenes and prevent the hydrogenation of butenes to butane are of great interest. It has been proposed that the observed product distribution is controlled by the adsorption energy of butadiene to the catalytic surface and weaker binding is known to direct the product distribution in favour of the formation of butenes^27^,^28^. Our surface science experiments show that Cu binds butadiene weaker than Pt, but the dissociation of molecular H_2_ on Cu surfaces and nanoparticle (NP) systems is often the rate limiting step. We use high-resolution microscopy to interrogate the atomic geometry of Pt/Cu alloys and discovered the minimum ensemble of Pt, a single atom, embedded in Cu(111) single crystal surface will dissociate H_2_ thus enabling selective hydrogenation reactions. Our surface science experiments reveal that isolated Pt atom geometries, unlike continuous Pt ensembles, exhibit weak binding of CO and maintain activity after many hydrogenation cycles. Guided by these model catalyst experiments, we prepare highly diluted Pt/Cu NPs using this single-atom alloy (SAA) principle^12^,^13^. NP Cu catalysts, with Pt as the minority species, are found to exhibit high activity and selectivity for butadiene hydrogenation to butenes under relatively mild conditions.

## Results

### Hydrogen uptake and desorption from Pt/Cu(111) surfaces

**Figure 1:**
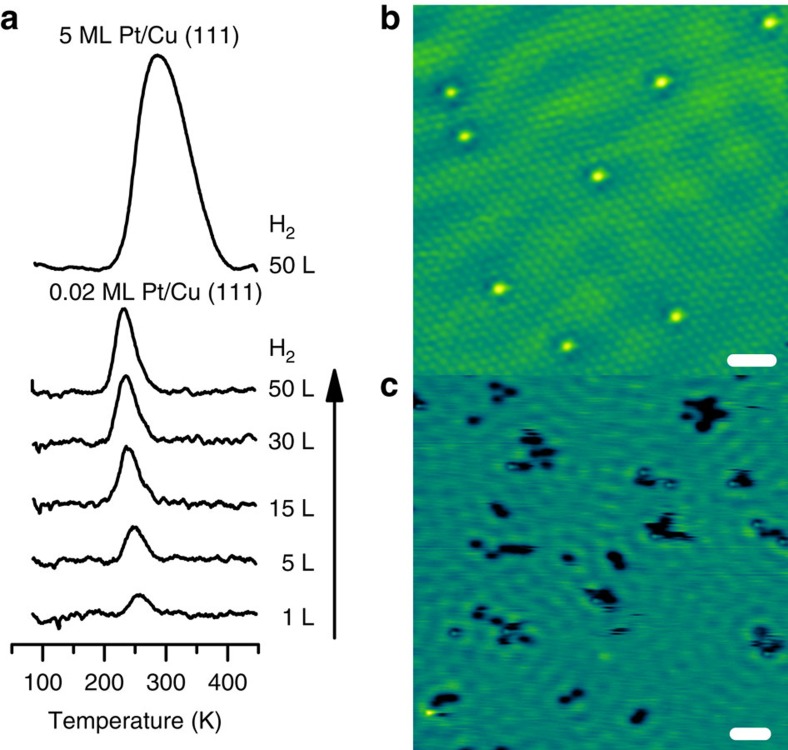
Hydrogen dissociation and spillover on a Pt/Cu(111) SAA. (**a**) TPD traces of H_2_ uptake on 0.02 ML and 5 ML Pt/Cu(111) surface exposed to H_2_ at 85 K. Gas exposures are quoted in Langmuirs (L). (**b**) STM image of 0.02 ML Pt/Cu(111) SAA surface where the Pt atoms appear as isolated protrusions substituted into the Cu(111) surface. Scale bar, 1 nm. (**c**) STM image showing H atom spillover onto Cu in which the H atoms appear as depressions and cluster into small mobile islands. Pt sites appear in STM images as protrusions. Scale bar, 3 nm.

Model Pt/Cu surfaces with a range of Pt coverages were prepared using physical vapour deposition of Pt onto a clean Cu(111) surface held at 380 K. Scanning tunnelling microscopy (STM) imaging (Fig. 1b) shows that at low Pt coverages (0.02 monolayer (ML)), Pt atoms exist as individual, isolated species substituted in the Cu surface lattice, which we refer to as a SAA^19^. The STM images indicate that Pt atoms incorporate both directly into Cu(111) terraces and in areas above surface step edges via place exchange. Even in regions of higher local concentration near step edges, the Pt atoms are always isolated from one another and do not form dimer or trimer clusters. This high dispersion of individual Pt atoms is driven by the negative mixing enthalpy of Pt in Cu and elastic strain relief of incorporating a larger Pt atom into a smaller Cu lattice.^15^

By quantifying surface coverages of both Pt and H, our temperature-programmed desorption (TPD) experiments (Fig. 1a) reveal that trace amounts of Pt make H available on the more inert Cu surface by dissociating H_2_ and allowing spillover of H atoms to Cu sites. Importantly, the H_2_ desorption temperature on the Pt/Cu(111) SAA of 230 K is significantly lower than H_2_ desorption from either a Pt(111) or Cu(111) surfaces, which occurs at ∼300 K (refs 29, 30, 31), meaning that the H atoms also recombine and desorb from the Pt atom sites. This is consistent with the principle of microscopic reversibility that states the forward and reverse reactions follow the same lowest energy pathway. With increasing exposure to H_2_, the desorption temperature shifts to lower temperatures characteristic of second-order desorption kinetics. Therefore, the Pt atoms in Cu act as both entrance and exit sites for H_2_ dissociation and recombination, respectively. Calculations predict that Pt exhibits a negligible barrier for H_2_ dissociative adsorption, which is consistent with the facile dissociative adsorption of H_2_ that we observe at 85 K (ref. 32). Notably, the concentration of H_2_ desorbing from the surface is much higher than the Pt coverage, demonstrating that the H atoms must spill over from the Pt sites to Cu. Low-temperature STM (LT-STM) allows us to directly image these H atoms. STM images of Pt/Cu(111) SAA surfaces exposed to H_2_ show mobile depressions on the Cu surface that Jewell *et al.*^33^ previously assigned as H atoms diffusing across a Cu surface. The presence of H atoms on Cu(111) demonstrates that H atoms do indeed spill over onto the Cu surface (Fig. 1c). This spillover process allows for an increase in weakly bound H on Cu (∼20 kJ mol^−1^; ref. 34), which is a prerequisite for selective hydrogenation chemistry.

The low-temperature desorption of H_2_ from the Pt/Cu(111) SAA surface is a result of a decrease in the H_2_ recombination barrier as compared with a Cu surface and a decrease in H atom binding energy compared with a pure Pt surface. These experimental results are consistent with theoretical studies that predict a minimal activation energy (<0.05 eV) for H_2_ dissociation at individual, isolated Pt sites in Cu and much weaker binding of H atoms to the Cu surface as opposed to pure Pt^32^,^35^. The weaker binding of H atoms at the single Pt atom dissociation sites compared with Pt(111) results from the preferential adsorption of H at threefold hollow sites, which in the SAAs are composed of one Pt atom and two Cu atoms. By combining complementary energetic landscapes for H_2_ dissociation and spillover, we later show that Pt/Cu bimetallic catalysts exhibit enhanced reactivity since Pt readily activates H_2_ and Cu exhibits weak binding of reaction intermediates. Thus, the Pt/Cu bimetallic combination addresses both the reduced selectivity of Pt catalysts and the barrier for H_2_ activation on Cu (refs 30, 36).

In addition, our experiments reveal that Pt/Cu(111) SAAs bind CO significantly weaker than Pt as evidenced by the lower desorption temperature of CO from single Pt atoms (350 K) compared with Pt(111) (ref. 32) (450 K) (Supplementary Fig. 1 and Supplementary Note 1). This result holds great promise for the utility of Pt/Cu SAAs given problems such as CO poisoning of the Pt anodes of PEM fuel cells^4^.

### Butadiene hydrogenation on Pt/Cu(111) alloys

**Figure 2:**
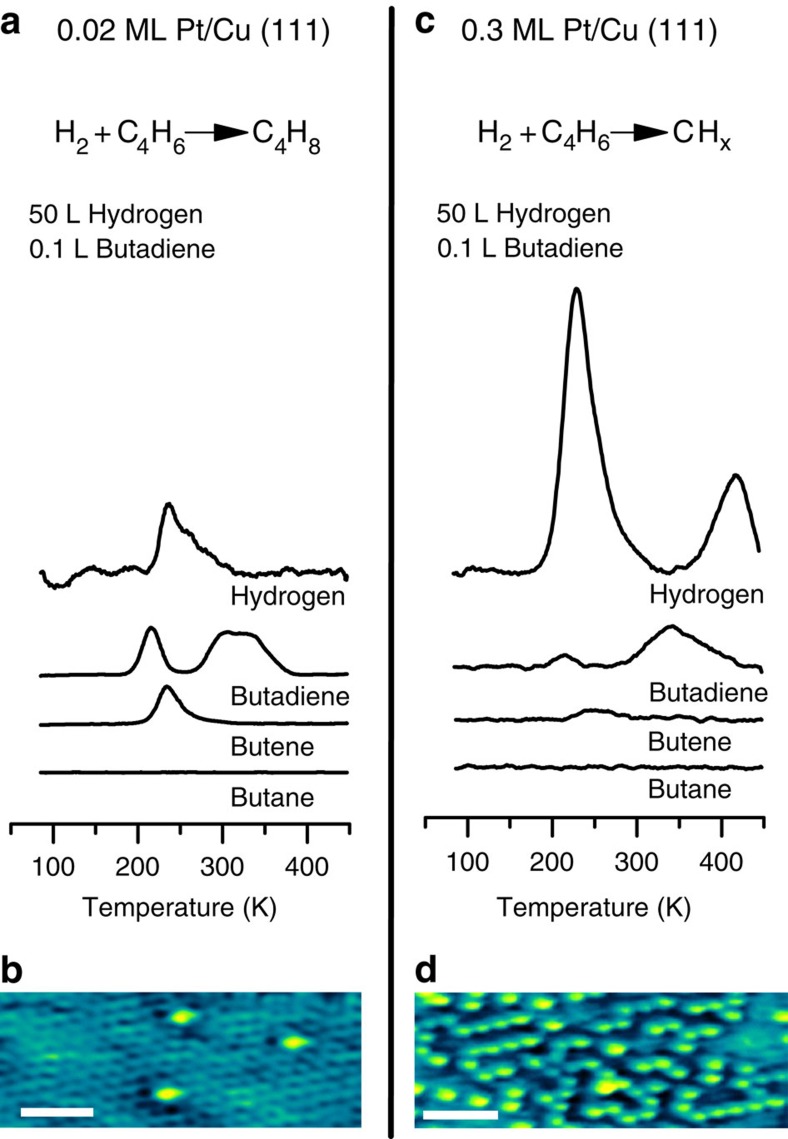
Selective hydrogenation of butadiene on Pt/Cu(111) SAAs versus larger Pt ensembles. TPD/R traces from the adsorption of H_2_ and butadiene on (**a**) 0.02 ML Pt/Cu(111) SAA and (**c**) 0.3 ML Pt/Cu(111). STM image of (**b**) 0.02 ML and (**d**) 0.3 ML Pt/Cu(111). Scale bars, 1 nm.

To probe the chemical reactivity of Pt/Cu SAAs, we performed TPD/reaction (TPD/R) studies on the model catalyst systems. Figure 2a shows TPD/R traces resulting from adsorption of 50 L H_2_ (which results in 0.2 ML of adsorbed H atoms (H_a_)) and 0.1 L butadiene (0.2 ML) on a 0.02 ML Pt/Cu(111) SAA. The sole product of co-adsorption of H_a_ and butadiene is butenes at 25% conversion with no detectable yield of butane. The hydrogenation reaction is facile on the SAA as evidenced by reactively formed butenes desorbing from the surface below room temperature (240 K). Unreacted butadiene desorbs from Cu terraces (220 K), Pt sites (290 K) and Cu steps (330 K), and unreacted H_2_ desorbs from Pt sites (230 K) (Supplementary Figs 2 and 3, and Supplementary Note 2). The desorption temperature of these unreacted species is consistent with desorption from a clean Pt/Cu(111) surface, demonstrating that the co-adsorption of butadiene and H_a_ does not alter the desorption behaviour of either species. In the absence of H_a_, butadiene reversibly adsorbs and desorbs from the surface without decomposing or inducing self-hydrogenation to unwanted butane (Supplementary Fig. 3). Reactively formed butenes desorb at the same temperature as adsorbed 1-butene (Supplementary Fig. 4), indicating that the reaction is desorption rate limited and the selective hydrogenation must occur at a temperature below 240 K (refs 37, 38, 39). Desorption of butane is never observed and co-adsorption of H_a_ and 1-butene did not yield the fully hydrogenated product butane (Supplementary Fig. 5), indicating that weak binding to Cu enables selective hydrogenation to the desired product.

Increasing the concentration of Pt to 0.3 ML decreased the selectivity of the surface to butenes. With increased Pt content, we predominantly observe decomposition of hydrocarbons resulting in surface-bound hydrocarbon fragments as evidenced by desorption of H_2_ at higher temperatures (Fig. 2c)^40^. High-temperature desorption of H_2_ (420 K) is indicative of the decomposition of butadiene and butenes into surface CH_*x*_ fragments that further decompose to C and release additional H_2_ at higher temperatures as previously observed for decomposed butenes on Pd model catalysts^40^. STM images of 0.3 ML Pt/Cu(111) reveal the cause of this effect; in addition to single Pt atoms, the 0.3-ML Pt surface is composed of extended linear chains (2–10 atoms) of Pt atoms (Fig. 2d). Unlike individual Pt atoms, these larger Pt ensembles within the Cu matrix are capable of breaking C–C and C–H bonds that leads to decomposition and active site poisoning. These Pt sites exhibit reduced selectivity similar to Pt(111) on which the decomposition of butadiene is preferred over hydrogenation due to strong binding of butadiene to Pt(111) and facile decomposition^41^,^42^,^43^,^44^.

**Figure 3:**
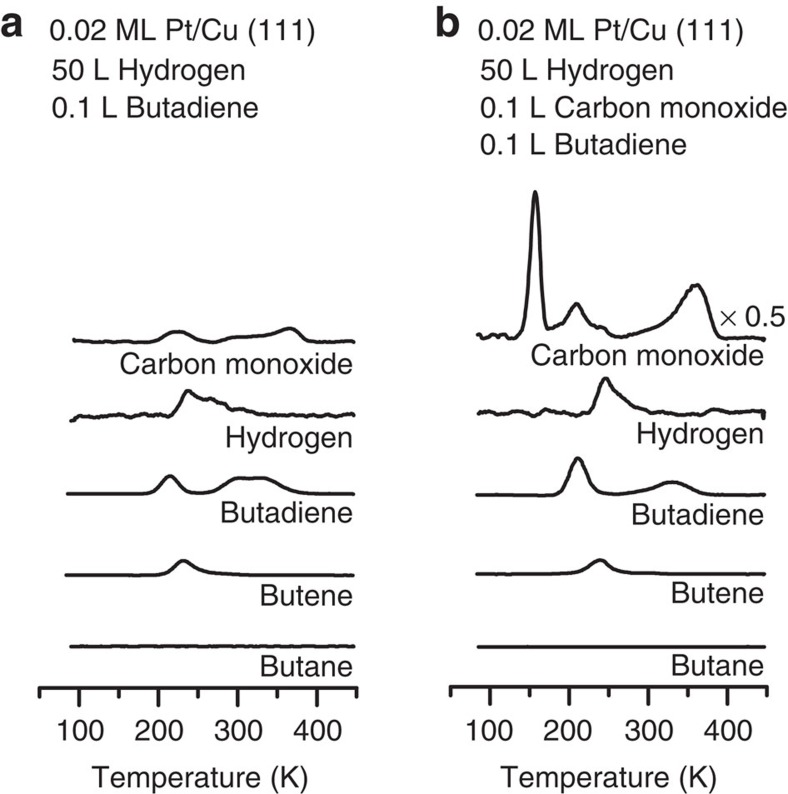
Butadiene hydrogenation in the presence of CO. TPD/R traces for the co-adsorption of H_2_ and butadiene (**a**) without and (**b**) with CO on 0.02 ML Pt/Cu(111). H_2_ was adsorbed on the surface before adsorption of CO.

To determine the active sites for hydrogenation, CO was used to selectively block Pt sites as the hydrogenation was performed. First, H was adsorbed onto a Pt/Cu(111) SAA and then small amounts of CO were adsorbed (Fig. 3). Owing to the stronger binding energy of CO to Pt than Cu, CO selectively adsorbs atop to the isolated Pt atoms^45^,^46^. H was adsorbed onto the surface before CO since CO blocks the adsorption of H. The surface was then exposed to butadiene and the temperature was ramped to perform the hydrogenation reaction. Since CO desorbs from Pt sites at higher temperature (350 K) than butenes or H_2_, CO inhibits any hydrogenation from occurring at the Pt sites. CO also blocks the adsorption of butadiene at Pt sites as seen by the disappearance of its desorption peak at 290 K. Our results indicate that the conversion of butadiene to butenes is unaffected by the CO adsorption at the Pt atom sites, meaning that the selective hydrogenation of butadiene occurs predominately on the Cu sites.

**Figure 4:**
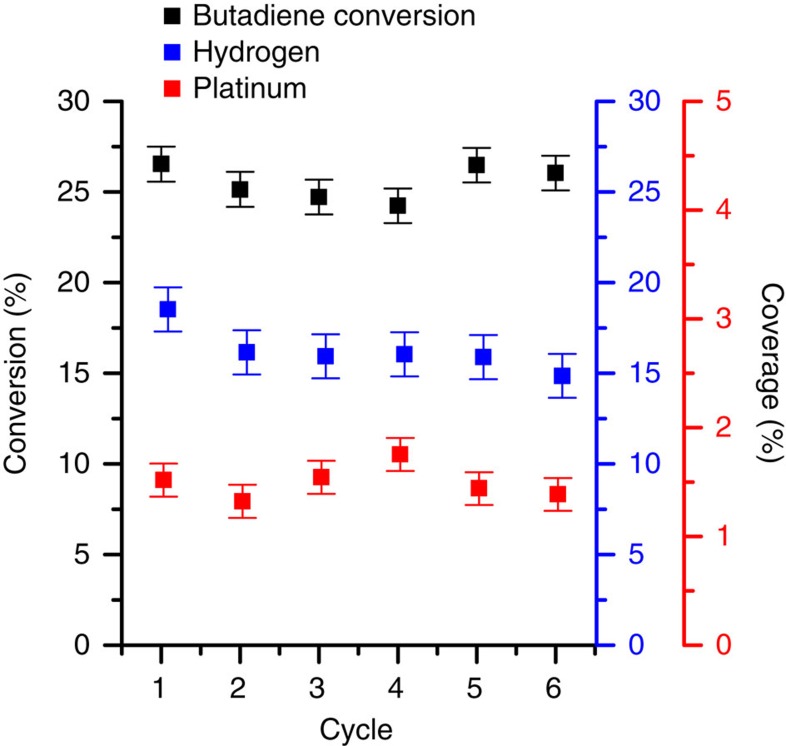
Repeated cycles of butadiene hydrogenation on a Pt/Cu(111) SAA reveal no loss in activity. Multiple cycles of TPD/R after the co-adsorption 50 L H_2_+0.1 L butadiene, followed by TPD of 50 L H_2_ and then TPD of 1 L CO on 0.02 ML Pt/Cu(111) SAA. Multiple co-adsorptions of H_2_ and butadiene lead to no decrease in the amount of butenes produced. The Pt atom concentration in the surface after each reaction cycle was quantified by CO titration. In each reaction cycle, the temperature was ramped from 85 to 450 K. Error bars are 1 s.d. of the data.

Pt/Cu(111) SAAs exhibit excellent stability after multiple TPD/R cycles as shown by the steady and selective conversion of butadiene to butenes. Cycles of the co-adsorption of hydrogen and butadiene followed by CO titration and H_2_ uptake showed no change in reactivity (Fig. 4). After six cycles, the conversion of butadiene to butenes remained constant at 25±1% as determined by the amount of butenes desorbing at 240 K. The number of Pt atoms in the surface was also quantified via CO titration between each reaction. After each TPD/R cycle, the concentration of Pt atoms in the surface layer was 1.4±0.2%, consistent with the number of Pt atoms present prior to each hydrogenation reaction. Since the Pt atoms serve as the entrance routes of H_a_ onto the surface, the ability for the surface to uptake H_a_ remained constant at 15±1%. Loss of Pt sites or decrease in H_2_ activation was not observed because Pt atoms are neither poisoned by decomposition of hydrocarbon fragments nor lost by sub-surface migration. Since multiple hydrogenation runs result in no decrease in activity or selectivity, single isolated Pt atoms in Cu are capable of H_2_ activation and spillover but not of breaking C–C bonds, thus preventing the butadiene decomposition and surface poisoning that occur at higher Pt coverage. The robust nature of the Pt/Cu system further highlights the durability of SAAs.

### Pt/Cu SAA NPs

Inspired by the performance and stability of highly dispersed Pt atoms in the model catalyst system, we applied our SAA strategy to prepare Pt/Cu bimetallic NPs and examined them at atmospheric pressure under several realistic reaction conditions. Three different Pt/Cu NP compositions were prepared by the galvanic replacement (GR) method^47^, in which a controlled amount of Pt was exchanged with Cu on pre-formed Cu NPs supported on γ-Al_2_O_3_. Low concentrations of Pt in solution form SAA Pt_0.1_Cu_14_/Al_2_O_3_ and Pt_0.2_Cu_12_/Al_2_O_3_ NPs, whereas higher concentrations of Pt form Pt_2_Cu_6_/Al_2_O_3_. A detailed description of the synthesis and characterization of all Pt/Cu alloy NPs is given in Supplementary Figs 6–14, Supplementary Tables 1 and 2, and Supplementary Note 3.

**Figure 5:**
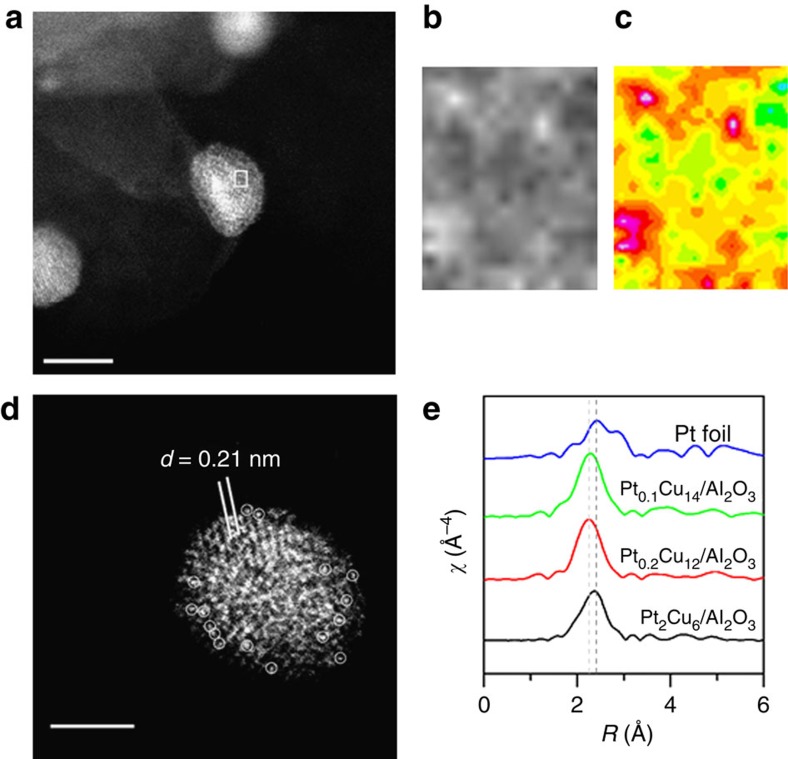
Characterization of Pt/Cu SAA NPs. (**a**–**d**) HAADF-STEM images with (**c**) coloured intensity map from selected region, and (**e**) EXAFS *k*^3^-weighted Fourier transforms. (**a**,**d**) Typical regions of the sample Pt_0.1_Cu_14_/Al_2_O_3_, showing Cu metal particles with isolated Pt atoms. Isolated Pt atoms are highlighted with circles. The lattice spacing of Cu is 0.21 nm. Scale bars, 5 nm (**a**) and 2 nm (**d**). (**b**) Enlarged image and (**c**) colorized intensity map of highlighted region showing isolated Pt atoms. (**e**) EXAFS data were collected at Pt-L_III_ edge at room temperature from Pt foil and in H_2_ atmosphere at Pt-L_III_ edge at room temperature from pre-reduced Pt_0.1_Cu_14_/Al_2_O_3_, Pt_0.2_Cu_12_/Al_2_O_3_ and Pt_2_Cu_6_/Al_2_O_3._

Elemental mapping by energy dispersive X-ray spectroscopy (EDS) reveals that the Pt/Cu NPs are all bimetallic. Pt, Cu, Al and O elemental mapping by EDS shows that Pt is distributed over the Cu NPs and is not deposited on the γ-Al_2_O_3_ support (Supplementary Fig. 10). *In situ* extended x-ray absorption fine structure (EXAFS) performed at room temperature at the Pt-L_III_ edge reveals the coordination of Pt in Pt/Cu alloys (Fig. 5e and Supplementary Table 2). No Pt–Pt bonds are detected in Pt_0.1_Cu_14_/Al_2_O_3_ and Pt_0.2_Cu_12_/Al_2_O_3_, which provides direct evidence for individual, isolated Pt atoms in the Pt/Cu bimetallic NPs. The Pt/Cu first-shell interaction distance is 2.63 Å, which is between Pt–Pt (2.77 Å) and Cu–Cu bond lengths (2.56 Å; ref. 48), further supporting SAA formation. The existence of Pt–Pt bonds in Pt_2_Cu_6_/Al_2_O_3_ suggests that Pt islands and/or clusters are formed in the NPs with this higher Pt loading.

Figure 5 shows aberration-corrected high-angle annular dark-field scanning transmission electron microscope (HAADF-STEM) images of Pt_0.1_Cu_14_/Al_2_O_3_. Aberration-corrected HAADF imaging can distinguish isolated Pt atoms due to differences in *Z*-contrast^49^,^50^. In this work, we observed a number of bright, atom-sized features within the Cu lattice (Fig. 5). Our EXAFS results indicate that these features are isolated Pt atoms in Cu. In addition, the lattice spacing of Pt/Cu is comparable to the pure Cu lattice spacing, which also supports dilute dispersion of Pt atoms (Fig. 5d). Variations in the background structure of the Cu NPs make imaging of isolated atoms difficult, but in Fig. 5d the single Pt atoms are more apparent due to the uniform background^50^. STEM and EXAFS analyses demonstrate the formation Pt/Cu SAA NPs, which provides a new catalytic system to study selective hydrogenation reactions.

### Butadiene hydrogenation on SAA NPs

**Figure 6:**
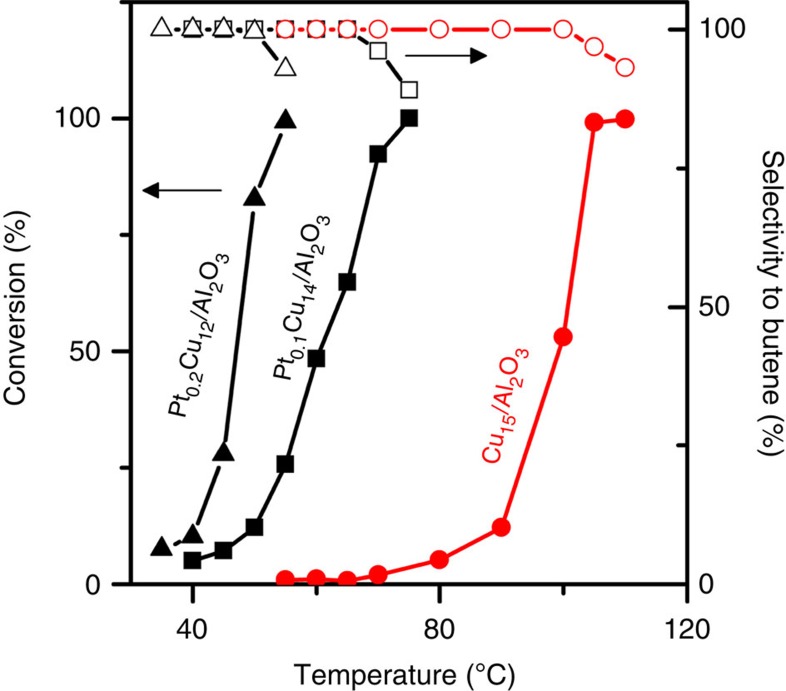
Selective hydrogenation of butadiene. Hydrogenation shown as a function of temperature over Cu_15_/Al_2_O_3_, Pt_0.1_Cu_14_/Al_2_O_3_ and Pt_0.2_Cu_12_/Al_2_O_3_ NPs (1,3-butadiene (1.25%), H_2_ (20%) and He (balance), GHSV=1,200 h^−1^).

Our catalytic data reveal that adding trace amounts of Pt to Cu NPs significantly enhances hydrogenation. The butadiene hydrogenation activity and selectivity as a function of temperature on Pt_0.1_Cu_14_/Al_2_O_3_, Pt_0.2_Cu_12_/Al_2_O_3_ and Cu_15_/Al_2_O_3_ NPs are shown in Fig. 6 and Supplementary Fig. 15. Under the conditions used, Pt_0.1_Cu_14_/Al_2_O_3_ has a hydrogenation reaction onset at 40 °C, which is 35 °C lower than that of the monometallic Cu catalyst. The reaction rate over the Pt_0.1_Cu_14_/Al_2_O_3_ at 60 °C is an order of magnitude higher than the monometallic Cu catalyst. Pt_0.2_Cu_12_/Al_2_O_3_ exhibits greater hydrogenation activity than Pt_0.1_Cu_14_/Al_2_O_3_ due to a higher Pt atom surface amount at the increased Pt loading. However, the selectivity remains unaffected because the Pt atoms continue to be isolated in Pt_0.2_Cu_12_/Al_2_O_3_, as found by EXAFS model fitting (Supplementary Table 2).

Notably, the SAA catalysts maintain the high selectivity to butenes exhibited by Cu. At full conversion, there is over 90% selectivity towards butene isomers. The selectivity of SAA NPs (Pt_0.1_Cu_14_/Al_2_O_3_ and Pt_0.2_Cu_12_/Al_2_O_3_) was comparable to Cu_15_/Al_2_O_3_, whereas Pt monometallic catalysts fully converted butadiene to butane under these conditions. Monometallic Pt is known for over-hydrogenating dienes and alkynes^51^,^52^. Therefore, by combining the hydrogen activation ability of Pt with the weak binding of butadiene on Cu and the latter's selectivity to butenes, Pt/Cu SAA catalysts exhibit superior performance for this important industrial reaction (Supplementary Figs 15–20 and Supplementary Note 4). Various other alloy surfaces, including Pd-Au and Sn-Pt, have been shown to improve selective hydrogenation reactions^53^,^54^. However, this is the first report that single Pt atoms can enhance selective hydrogenation reactions on a less active metal, such as Cu.

**Figure 7:**
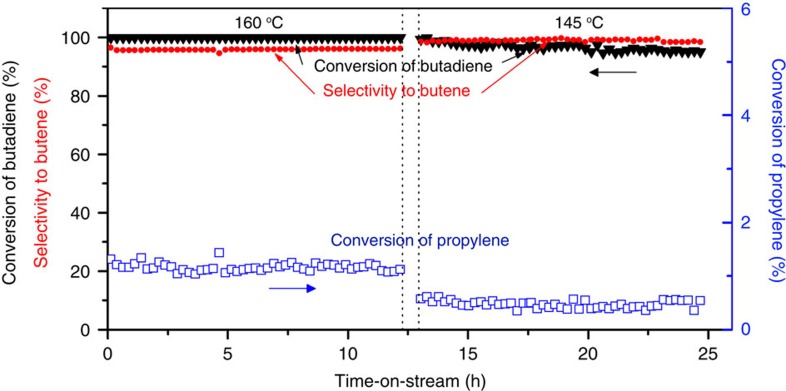
Butadiene conversion in the presence of excess propylene. Conversion and selectivity in long-time steady-state selective hydrogenation of butadiene at 160 and 145 °C (∼0.1 g catalyst Pt_0.1_Cu_14_/Al_2_O_3_, flow rate=50 ml min^−1^, 2% 1,3-butadiene, 20% propylene, 16% H_2_ and balance He. GHSV=12,000 h^−1^).

To demonstrate the ability of Pt/Cu SAAs to selectively hydrogenate alkadiene impurities in alkene feedstocks^25^, we tested the selective hydrogenation activity of Pt_0.1_Cu_14_/Al_2_O_3_ catalysts in the presence of excess propylene and found that butadiene is preferentially hydrogenated on the Pt/Cu SAA NPs (Supplementary Fig. 19). Below 120 °C, conversion of propylene was not observed. At 100% conversion of butadiene, <1% of propylene was converted to propane. Comparing these results with the hydrogenation activity of Pt_0.1_Cu_14_/Al_2_O_3_ in propylene-free condition, we found that the propylene has no effect on the activity and selectivity of Pt/Cu SAAs for the hydrogenation of butadiene. At 160 and 145 °C, butadiene is fully converted and >95% converted, respectively, over Pt_0.1_Cu_14_/Al_2_O_3_ without significant propylene hydrogenation (1.2% at 160 °C and 0.5% at 145 °C) for >12 h on-stream at each temperature (Fig. 7) demonstrating high stability of the catalysts at full conversion of butadiene.

Pt/Cu SAAs exhibit high stability and selectivity under realistic hydrogenation conditions (Supplementary Figs 16–20). The Pt/Cu SAAs maintain stable butadiene conversion for >46 h at 160 °C (Supplementary Fig. 16). Temperature-programmed oxidation studies of the catalysts after hydrogenation show negligible CO_2_ formation (Supplementary Fig. 20) demonstrating the robustness of SAA catalysts to butadiene decomposition, oligomerization and coke formation. Based on our model catalyst studies, the increase in the selectivity of SAAs is due to the inhibition of hydrocarbon decomposition commonly observed with Pt catalysts because SAAs do not offer extended Pt ensembles where these unfavourable reactions occur. In addition, it is known that Cu or Pd catalysts for diene and alkyne hydrogenation are affected by oligomer formation at mild temperatures, which leads to instability in their hydrogenation activity^55^. By running the reaction at higher temperatures, we do not observe any instability in the catalysts due to oligomerization (Supplementary Fig. 16). We found that after performing the reaction at near ambient temperatures, the hydrogenation activity declines due to adsorption of hydrocarbon species. However, the activity can be fully recovered by heating in H_2_ at 350 °C, which effectively desorbs the hydrocarbons (Supplementary Fig. 17). Running at the higher temperatures shown in Fig. 7 preserved the catalyst activity without affecting its selectivity.

## Discussion

In this work, surface science and atomic resolution microscopy studies of the selective hydrogenation of butadiene to butenes on model Pt/Cu(111) alloy surfaces have shown that individual, isolated Pt atoms in the Cu(111) surface are all that is required to ensure stable activity and 100% selectivity. We have extended these findings to realistic pressures (1 bar) by synthesizing and testing Pt/Cu SAA NP catalysts. At low loadings, Pt exists as individual, isolated atoms substituted into the Cu(111) surface. These single Pt atoms activate the dissociation and spillover of H to Cu. The weak binding of butadiene to Cu allows for the highly selective hydrogenation to butenes. No decomposition or poisoning of these alloys was observed, which can be attributed to the lack of extended Pt surface sites. At higher Pt content in Pt/Cu alloy surfaces, we directly visualized extended Pt ensembles responsible for the reduced selectivity. Isolated Pt atoms also bind CO significantly more weakly than metallic Pt, which is an important consideration in many Pt-catalysed reactions. This combined model system/NP catalyst strategy is a powerful approach to the design of new alloy catalysts. This approach is especially useful for highly diluted single-site systems in which identification of the active sites and elucidation of the surface chemistry is very challenging. Furthermore, this work reveals that, in addition to their promising selective hydrogenation properties, SAAs provide the ultimate limit for the most efficient use of costly catalytic elements such as Pt.

## Methods

### Ultra-high vacuum studies

STM and TPD/R experiments were performed in three separate ultra-high vacuum chambers with base pressures <1 × 10^−10^ mbar. Cu(111) single crystals were clean with cycles of Ar^+^ sputtering (1.5 keV, 15 μA) and annealing to 700 K. A flux-monitored EFM 3 electron beam evaporator (Focus GmbH) was used to deposit Pt at a flux of 0.02 ML per min onto Cu(111) held at 380 K. STM images were acquired on a variable-temperature STM and a LT-STM (Omicron NanoTechnology) with the sample held at 30 or 5 K, respectively. Atomic resolution was obtained at tunnelling conditions of 150–200 nA and biases between 0.01 and 0.05 V. Pt/Cu(111) samples were exposed to 20 L H_2_ (99.9% Airgas) at 80 K and then cooled to 5 K for imaging with LT-STM. STM images of H adatoms were obtained at non-perturbative conditions of 30 pA and 30 mV. TPD traces were obtained in a chamber equipped with a quadrupole mass spectrometer. Pt/Cu(111) surfaces were cooled to 85 K with liquid nitrogen and exposed to H_2_ (99.9% Airgas), CO (99.99% Airgas), 1-butene (≥99% Aldrich) and/or 1,3-butadiene (≥99.5% Aldrich). Exposures are quoted in Langmuir (1 L=1 × 10^−6^ torr). TPD/R measurements were performed with a linear heat ramp 1 Ks^−1^. The integrated areas under the TPD traces are proportional to the number of molecules desorbing from the surface. H_2_ coverages were determined by the saturation coverage of (1 × 1) H adsorbed on 5 ML of Pt, assuming that the surface terminates as Pt(111).

### Synthesis and characterization

Pt/Cu NPs with different Pt loadings were synthesized using the GR method on the pre-reduced Cu NPs as described by Boucher *et al.*^13^ The Cu NPs were pre-formed and supported on γ-Al_2_O_3_ (ultra-pure grade 99.99%, surface area 70–100m^2^ g^−1^, Inframat Advanced Materials; heat-treated in air at 400°C), followed by calcination in air at 350°C. GR took place in aqueous solution under nitrogen protection with constant stirring and refluxing at 100 °C. Desired amounts of Pt precursor (H_2_PtCl_6_ · *x*H_2_O, Sigma-Aldrich) were added to a suspension of Cu NPs in an aqueous solution containing HCl (2 mM). After 20 min, the resulting material was filtered, washed and dried in vacuum.

Aberration-corrected HAADF-STEM images of Pt/Cu NPs were obtained at a nominal resolution of 0.07 nm using a JEOL 2200FS-AC STEM/TEM equipped with a hexapole corrector (CEOS GmbH, Heidelberg, Germany) at Oak Ridge National Laboratory. EDS imaging was conducted with a Bruker-AXS 30-mm^2^ silicon-drift detector system and Pt, Cu, Al and O elemental maps were collected. The instrument was operated at 200 kV for all imaging and EDS work.

X-ray absorption spectroscopy (XAS) measurements at the Pt-L_III_ edge were made at Argonne National Laboratory and Brookhaven National Laboratory in fluorescence mode at room temperature. All samples were reduced in H_2_ *in situ* before the measurements. Experimental and analysis details on the XAS are described in the Supplementary Methods.

### Catalytic activity measurements

The selective hydrogenation activity of the catalysts was tested in a quartz-bed flow reactor for 1,3-butadiene hydrogenation with 400 mg of catalyst diluted by 1.5 g of quartz particles. The as-synthesized Pt/Cu catalysts were reduced in H_2_ at 350 °C for 4 h before the reaction, the Cl residues were removed in H_2_ as well. A gas mixture of 1.25% 1,3-butadiene, 20% H_2_ and balance He (flow rate=20 ml min^-1^, Gas hourly space velocity (GHSV)=1,200 h^−1^) was introduced at 120 °C, followed by descending temperature testing. Gas chromatograph injections were done at each temperature after the temperature was stabilized for at least 10 min. The exit gas stream was analysed in a flame ionization detector (FID) of a HP6890 gas chromatograph system equipped with a 30-feet column (1/8 inches, filled with Sebaconitrile 20% Chromosorb Paw 80/100).

The activity tests with added propylene were conducted with a gas mixture of 2% 1,3-butadiene, 20% propylene, 16% H_2_ and balance He at a flow rate of 50 ml min^−1^ (100 mg catalyst, GHSV=12,000 h^−1^). After the activity tests with ascending temperature up to 170 °C (Supplementary Fig. 12), the long-time stability tests were performed isothermally at 160 and 145 °C for 12 h at each temperature.

## Additional information

**How to cite this article:** Lucci, F. R. *et al.* Selective hydrogenation of 1,3-butadiene on platinum–copper alloys at the single-atom limit. *Nat. Commun.* 6:8550 doi: 10.1038/ncomms9550 (2015).

## Supplementary Material

Supplementary Information Supplementary Figures 1-20, Supplementary Tables 1-2, Supplementary Notes 1-4, Supplementary Methods and Supplementary References
