# Supplementary material for: Selective hydrogenation of 1,3-butadiene on platinum–copper alloys at the single-atom limit
Source: Nat Commun. 2015 Oct 9;6:8550. doi: 10.1038/ncomms9550 (PMC4633827; doi:10.1038/ncomms9550)
Supplement: Supplementary Information — Supplementary Figures 1-20, Supplementary Tables 1-2, Supplementary Notes 1-4, Supplementary Methods and Supplementary References [file ncomms9550-s1.pdf]

Supplementary Figures

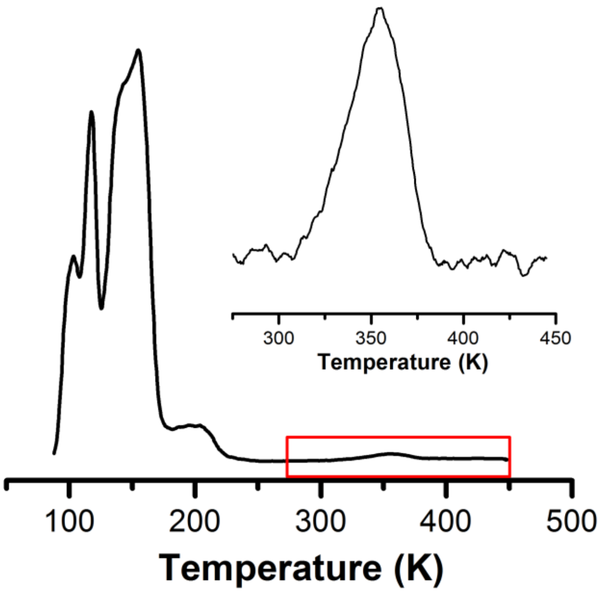

Supplementary Figure 1. CO TPD trace from 0.02 ML Pt/Cu(111) SAA exposed to 10 L CO. Inset shows a zoom in of the temperature range from 250 to 450 K where CO desorbing from Pt sites is observed.

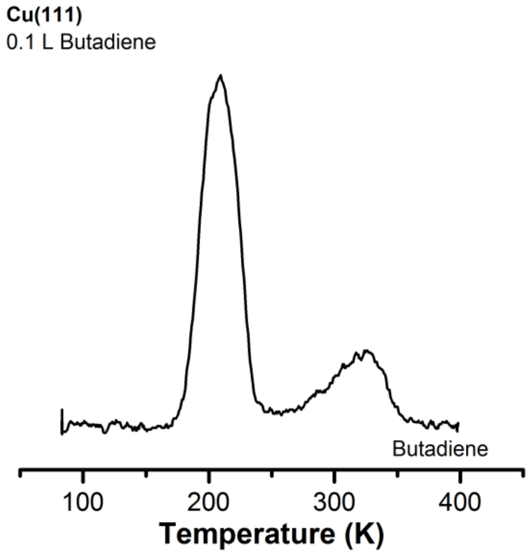

Supplementary Figure 2. Temperature programmed desorption traces for adsorption of 0.1 L butadiene on Cu(111) in the absence of H.

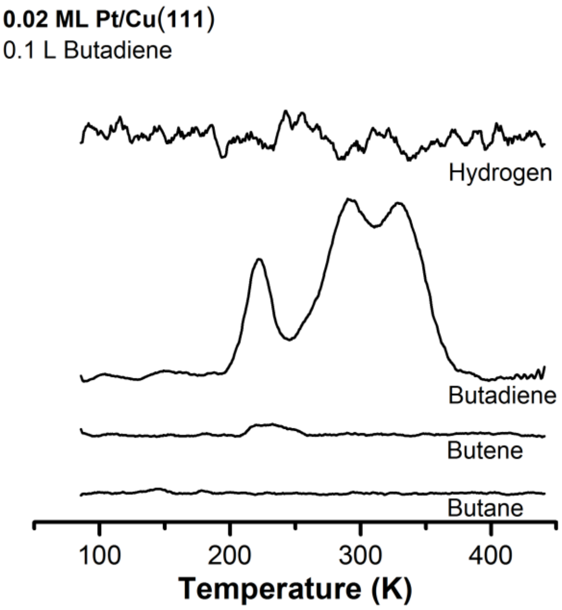

Supplementary Figure 3. Temperature programmed desorption traces for the adsorption of 0.1 L butadiene on 0.02 ML Pt/Cu(111) in the absence of H. Resulting desorption traces for hydrogen, butadiene, butenes, and butane.

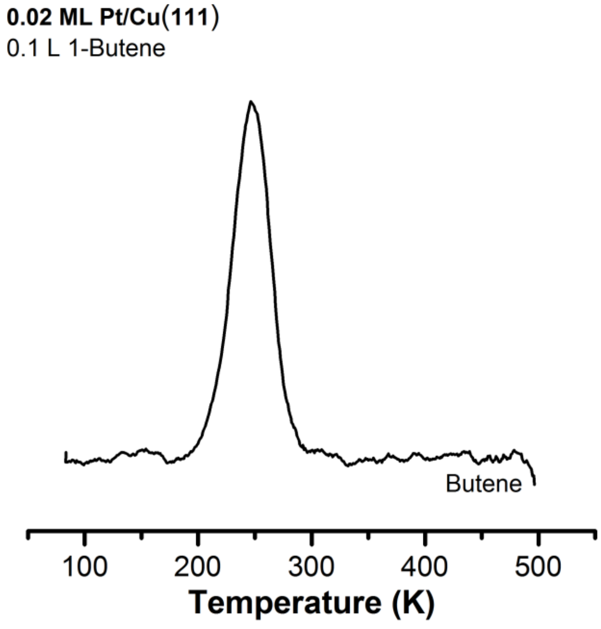

Supplementary Figure 4. Temperature programmed desorption trace for the adsorption of 1-butene on 0.02 ML Pt/Cu(111).

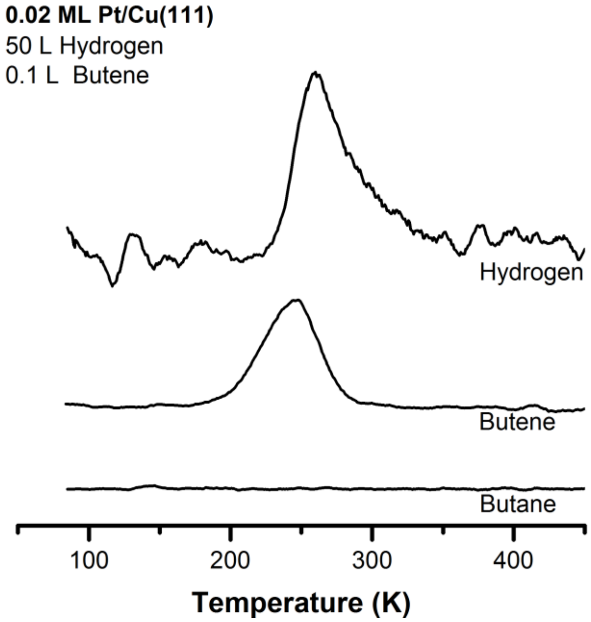

Supplementary Figure 5. Temperature programed desorption traces for the co-adsorption of 1-butene and hydrogen showing the lack of hydrogenation of 1-butene on 0.02 ML Pt/Cu(111). 50 L H_2 and 0.1 L 1-butene was adsorbed onto 0.02 ML Pt/Cu(111).

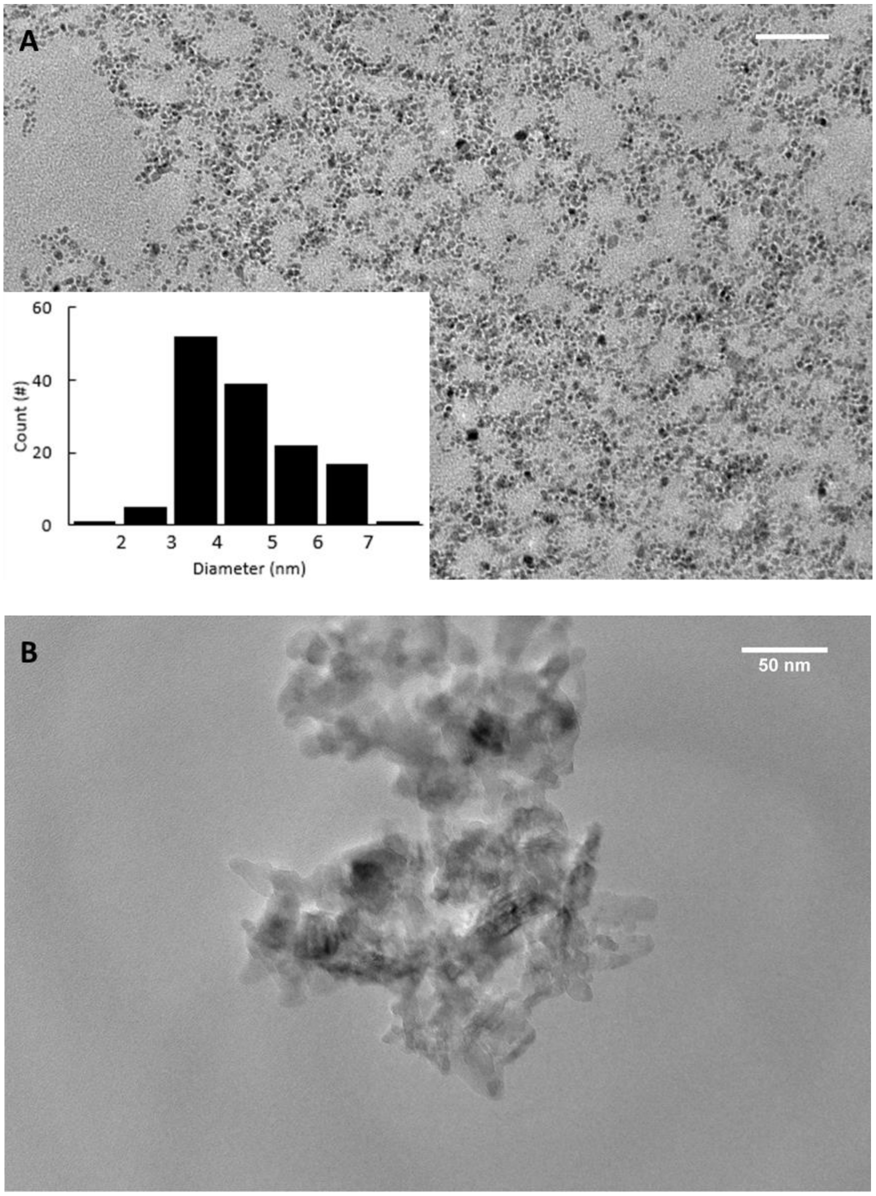

Supplementary Figure 6. (A) TEM images of unsupported Cu NPs, inset is particle size distribution (scale bar=50 nm). (B) TEM image of ϒ-Al_2O_3 supported Cu NPs (calcined).

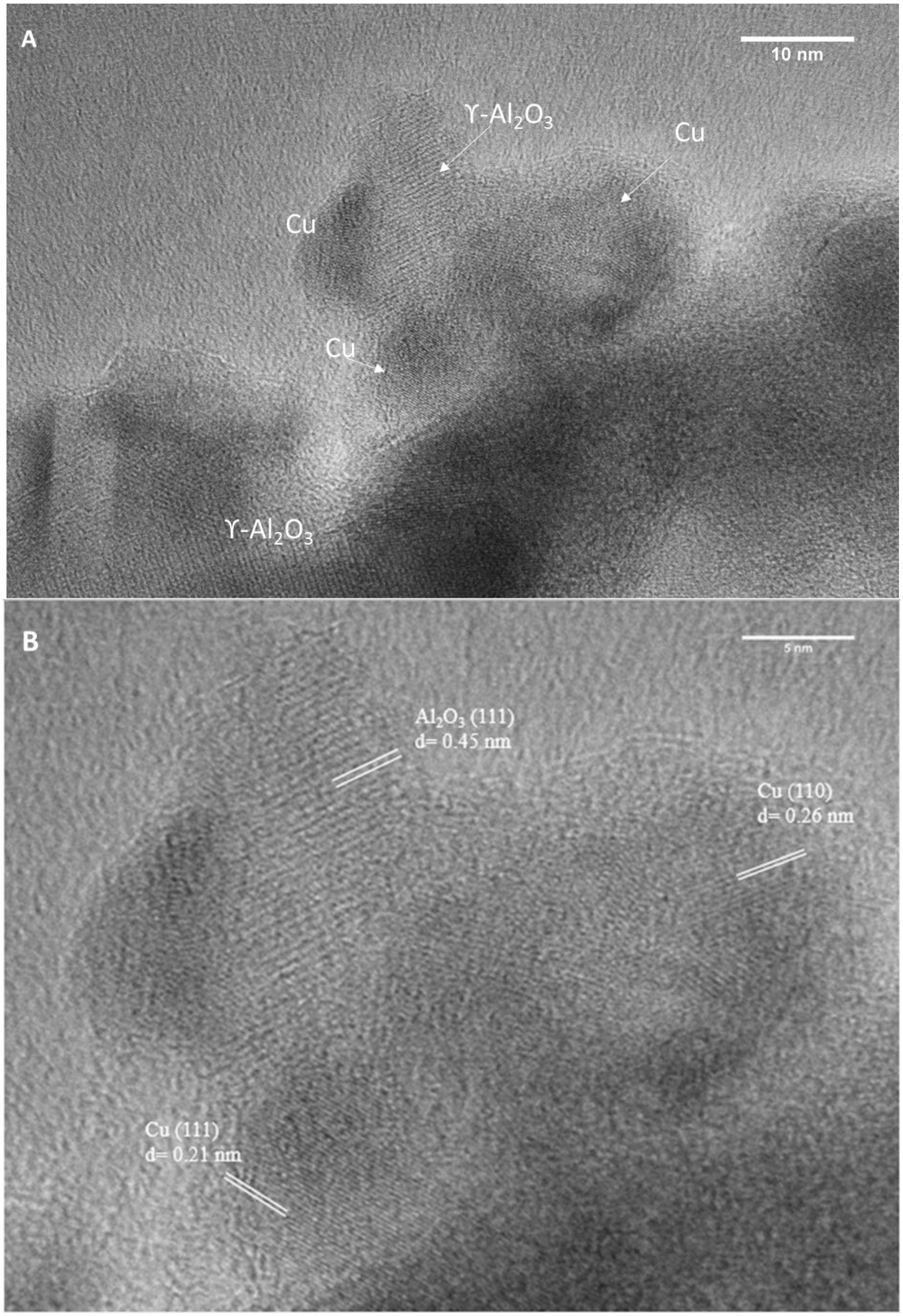

Supplementary Figure 7. (A) HR-TEM images of supported Cu NPS. (B) An enlarged region from (A).

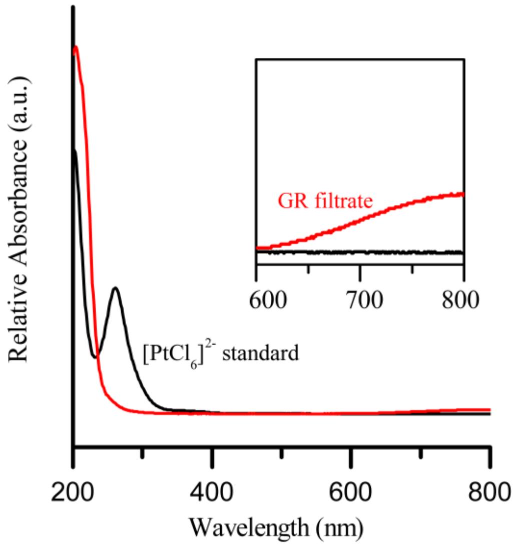

Supplementary Figure 8. UV-Vis spectra of $PtCl_6^{2-}$ standards compared to the filtrate from GR preparation of $Pt_{0.1}Cu_{14}/Al_2O_3$.

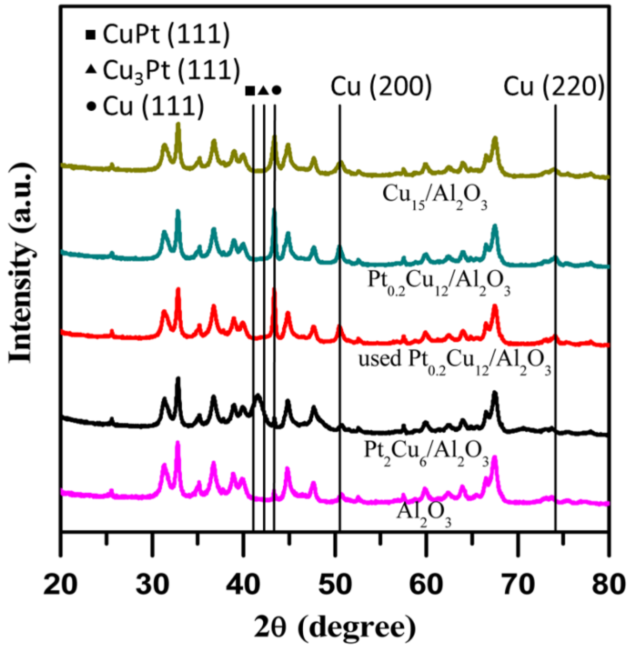

Supplementary Figure 9. XRD analysis of selected samples including a fresh and $Pt_{0.2}Cu_{12}/Al_2O_3$ used for butadiene hydrogenation. All catalysts, except the sample used in the hydrogenation reaction, were reduced in H_2 prior to XRD. (Cu JCPDS No. 85-1326, Cu_3Pt JCPDS No. 65-3247, CuPt JCPDS No. 48-1549)

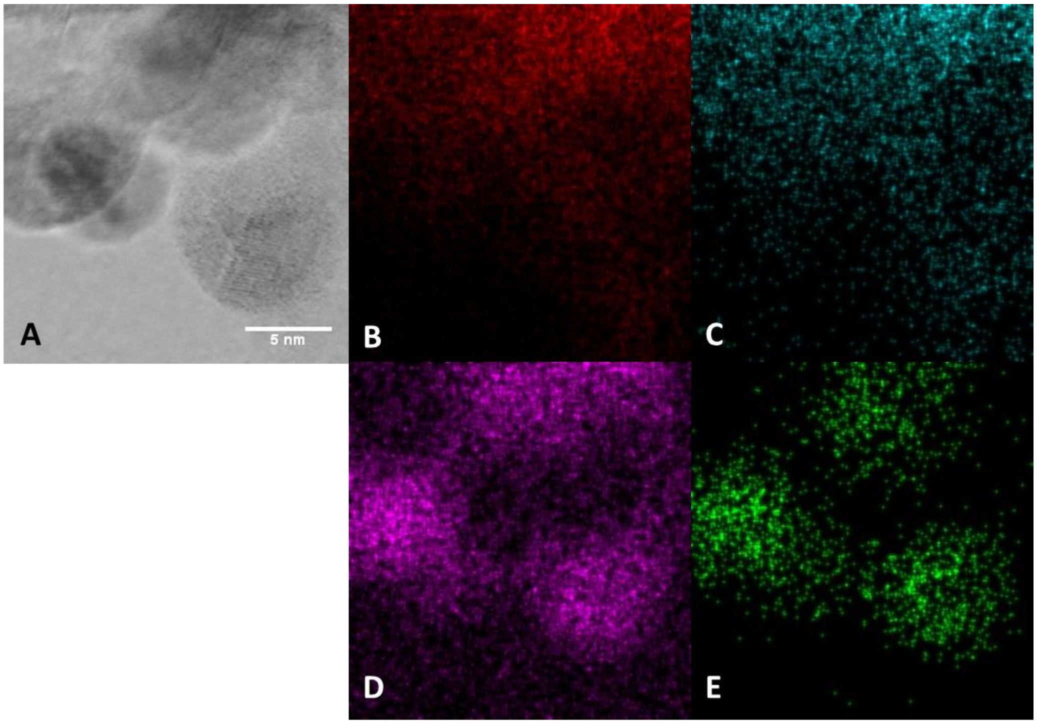

Supplementary Figure 10. (A) STEM bright-field image and elemental mapping (EDS) of $Pt_{0.1}Cu_{14}/Al_2O_3$. (B) Al, (C) O, (D) Cu and (E) Pt maps obtained from the same region on the sample.

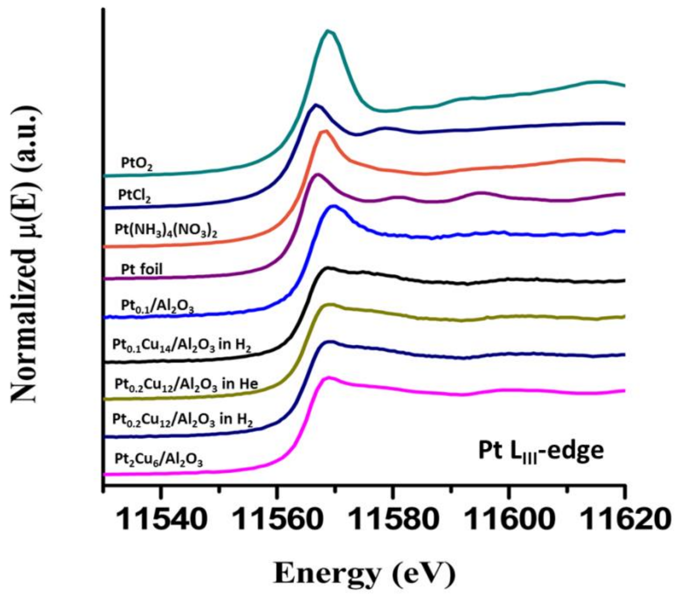

Supplementary Figure 11. Normalized *in situ* XANES spectra of selected samples collected at room temperature in H_2 (except where otherwise noted). The Pt/Cu samples were pre-reduced in H_2 at 350 °C *in situ*, while the $Pt_{0.1}/Al_2O_3$ was pre-reduced in H_2 at 400 °C *in situ.*

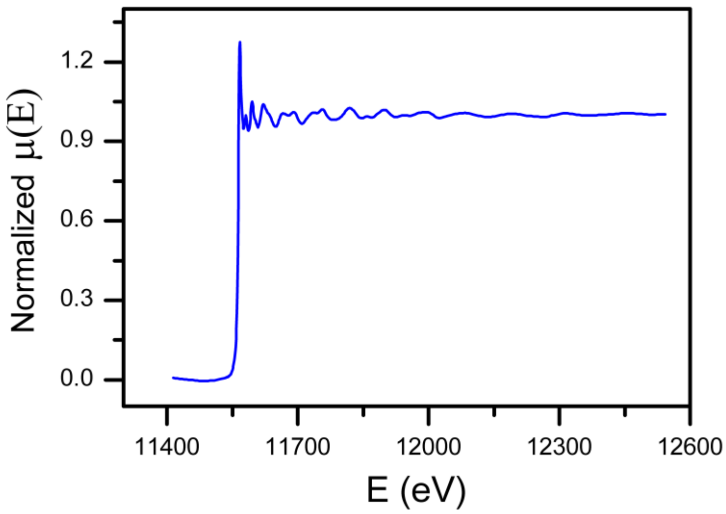

Supplementary Figure 12. Normalized absorption coefficient as a function of X-ray energy of a Pt foil.

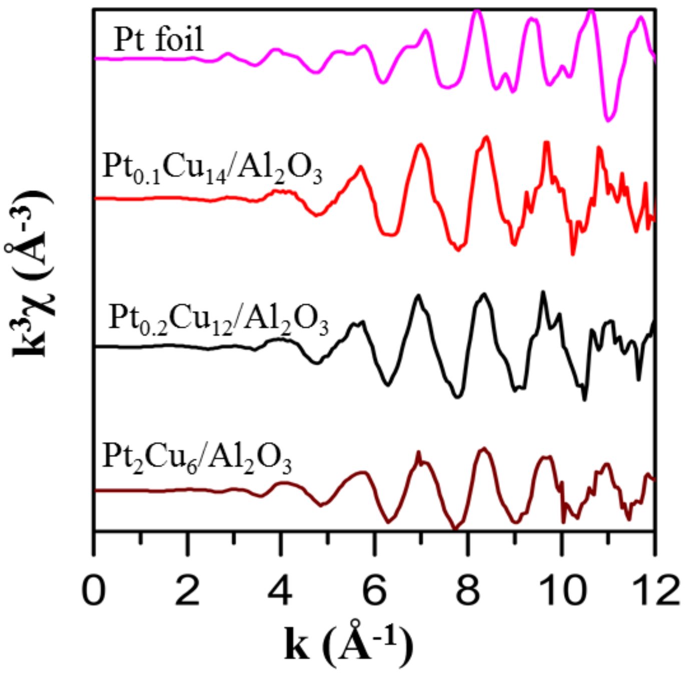

Supplementary Figure 13. *In situ* Pt L_{III} EXAFS of selected samples and Pt foil plotted in k space.

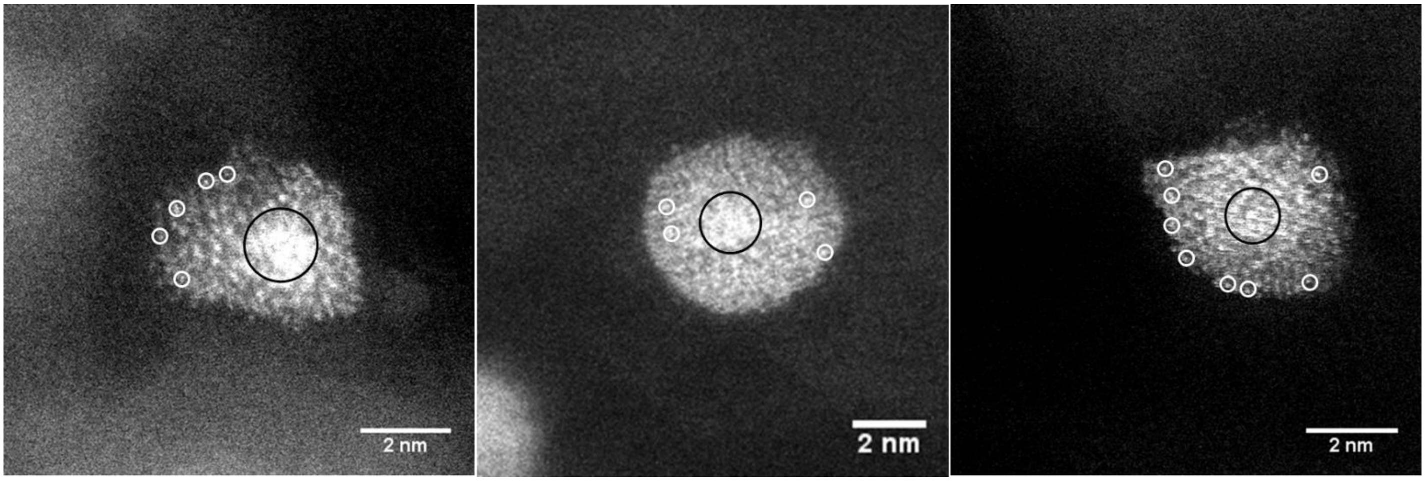

Supplementary Figure 14. Typical HAADF-STEM images of $Pt_{0.1}Cu_{14}/Al_2O_3$. The samples were pre-reduced in H_2 at 350 °C. Isolated Pt atoms are highlighted by white circles.

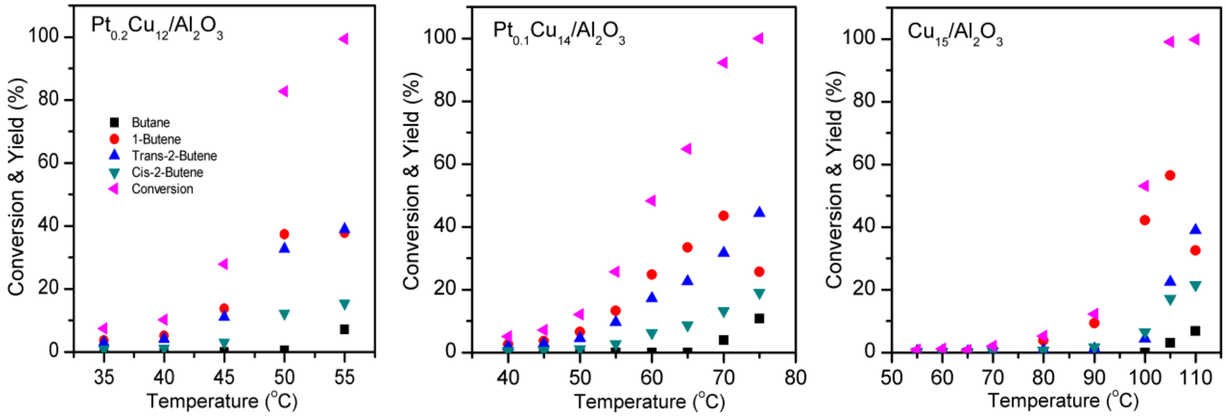

Supplementary Figure 15. Conversion and product yields in the selective hydrogenation of butadiene corresponding to Figure 6. (0.4 g catalyst, flow rate = 20 ml/min; 1, 3-butadiene (1.25 %), H_2 (20 %) and He (balance), GHSV=1,200 h^{-1})

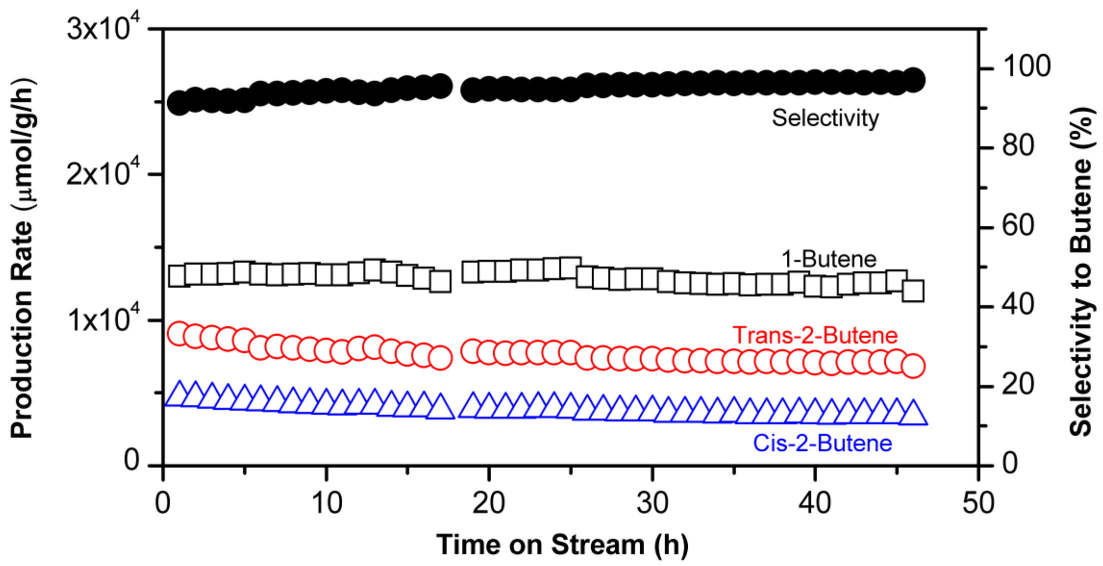

Supplementary Figure 16. Long-time hydrogenation steady-state activity test over $Pt_{0.1}Cu_{14}/Al_2O_3$ at 160 °C. (~ 0.1 g catalyst, flow rate = 50 ml/min. 2% 1, 3-butadiene, 20% H_2 and balance He. GHSV=12,000 h^{-1}. Conversion of butadiene in one hour is around 98 %).

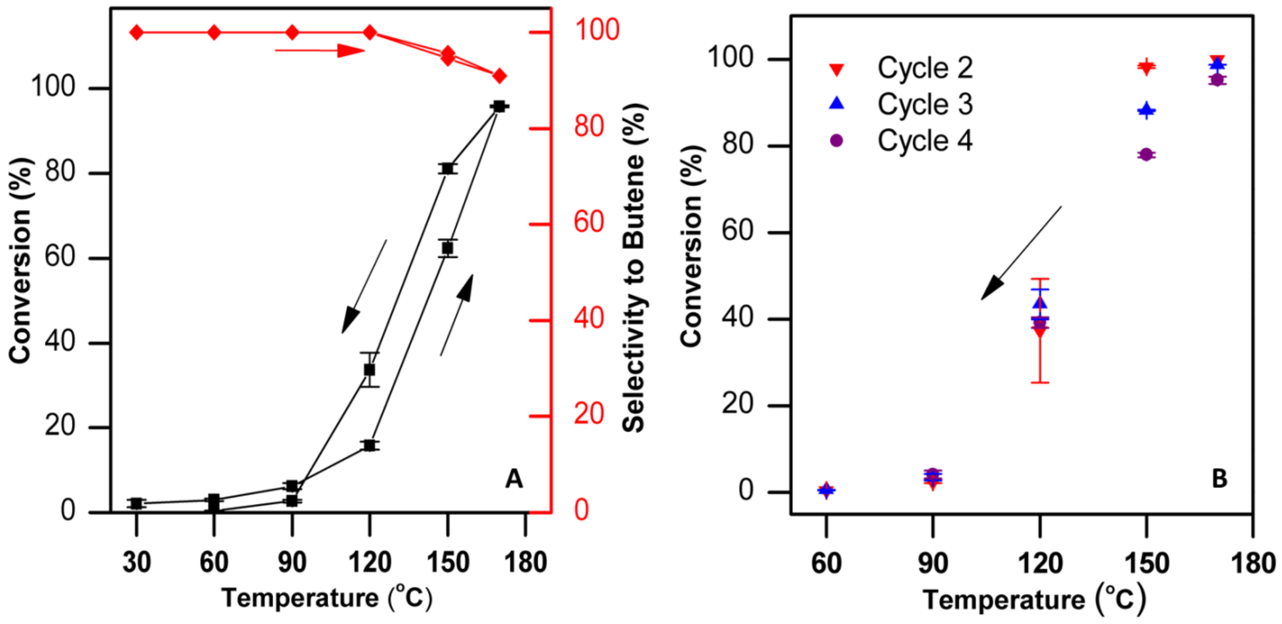

Supplementary Figure 17. Selective hydrogenation of butadiene as a function of temperature over $Pt_{0.1}Cu_{14}/Al_2O_3$. (0.1 g catalyst, flow rate = 50 ml/min. 2 % 1,3-butadiene, 20 % H_2 and balance He. GHSV=12,000 h^{-1}) (A) Conversion of butadiene and selectivity to butene with increasing and decreasing temperature ramps. (B) Conversion of butadiene in cyclic hydrogenation experiments, the catalysts were treated in H_2 at 350 °C between each cycle. The conversion of the first cycle is shown in (A).

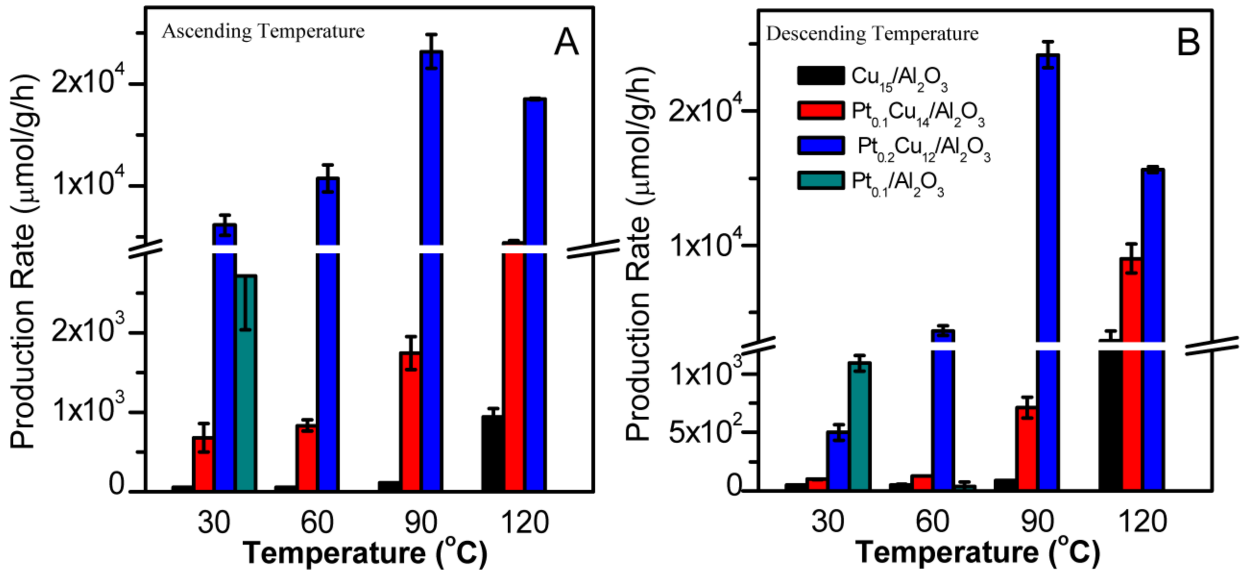

Supplementary Figure 18. Production rate of butenes over Cu_{15}/Al_2O_3, $Pt_{0.1}/Al_2O_3$, $Pt_{0.1}Cu_{14}/Al_2O_3$ and $Pt_{0.2}Cu_{12}/Al_2O_3$ at different temperatures. Shown are both (A) ascending and (B) descending temperature ramps to 120 °C.

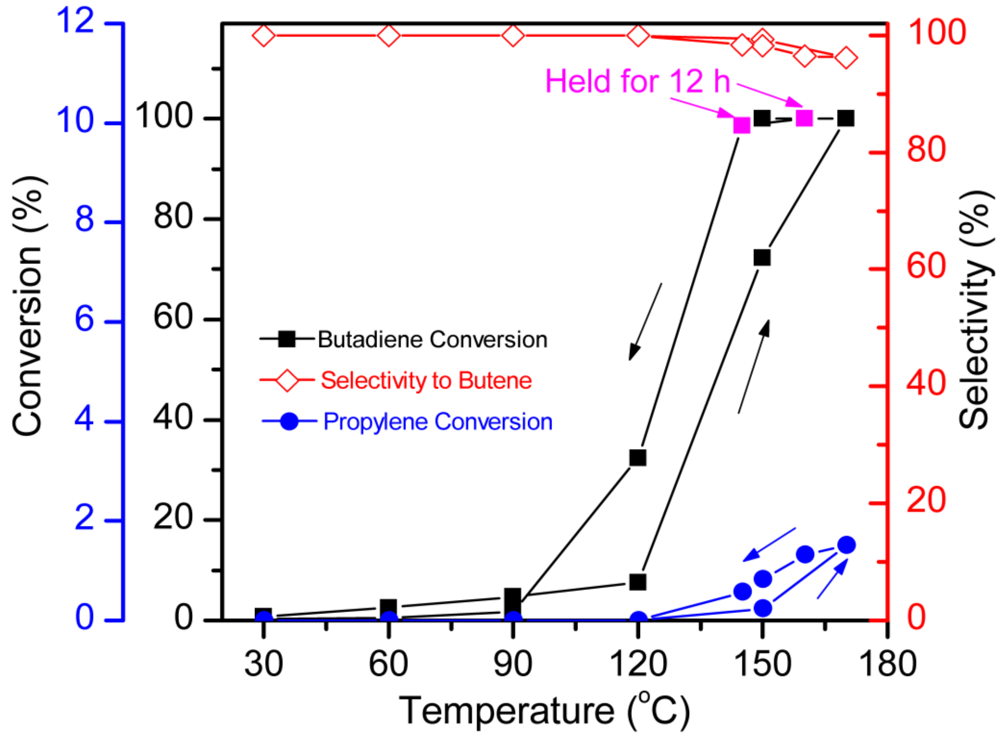

Supplementary Figure 19. Conversion and selectivity in the selective hydrogenation of butadiene over $Pt_{0.1}Cu_{14}/Al_2O_3$ as a function of temperature. (~ 0.1 g catalyst, flow rate = 50 ml/min. 2 % 1, 3-butadiene, 20 % propylene, 16 % H_2 and balance He. GHSV=12,000 h^{-1}).

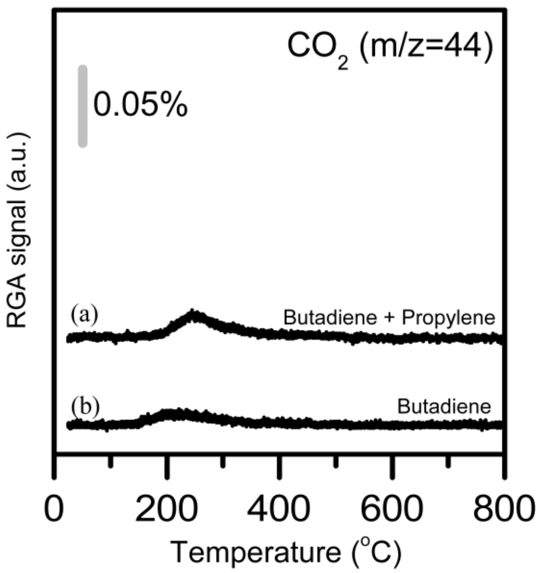

Supplementary Figure 20. TPO profile of spent $Pt_{0.1}Cu_{14}/Al_2O_3$ catalyst used for the long-time activity test in (a) 2 % 1, 3-butadiene, 20 % propylene, 16 % H_2 and balanced He for around 48 h; (b) 2 % 1, 3-butadiene, 20 % H_2 and balanced He for around 30 h. (TPO condition: 20 % O_2/He, 50 ml/min, 3 °C/min) Scale bar corresponds to 0.05 % CO_2 in the gas stream.

Supplementary Tables

Supplementary Table 1. Sample composition

**Sample**	**Composition**[a]	**Preparation Method**
Cu_{15}/Al_2O_3	14.9 at.% Cu	Colloidal
$Pt_{0.1}/Al_2O_3$	0.12 at.% Pt	IWI[b]
$Pt_{0.1}Cu_{14}/Al_2O_3$	0.11 at.% Pt, 13.7 at.% Cu	GR
$Pt_{0.2}Cu_{14}/Al_2O_3$	0.25 at.% Pt, 12.3 at.% Cu	GR
Pt_2Cu_6/Al_2O_3	2.2 at.% Pt, 5.6 at.% Cu	GR

[a] Determined by ICP-AES.
[b] Incipient wetness impregnation (IWI).

Supplementary Table 2. EXAFS model fitting

Sample	Shell	CN[a]	R[b] (A)	σ^2 (A^2)	R-factor
**Pt foil**	Pt-Pt	12	2.766 ± 0.003	0.0052	0.015
**$Pt_{0.1}Cu_{14}/Al_2O_3$**	Pt-Pt	0	--	--	0.02
	Pt-Cu	10.45	2.627 ± 0.007	0.0062	
**$Pt_{0.2}Cu_{12}/Al_2O_3$**	Pt-Pt	0	--	--	0.018
	Pt-Cu	10.1	2.631 ± 0.006	0.0063	
**Pt_2Cu_6/Al_2O_3**	Pt-Pt	2.55	2.695 ± 0.011	0.0023	0.01
	Pt-Cu	8.62	2.628 ± 0.006	0.0089	

[a] CN, coordination number; [b] R, distance between absorber and backscattered atoms. R-factor, closeness of the fit, if < 0.05, consistent with broadly correct models.

Supplementary Notes

Supplementary Note 1: Carbon monoxide desorption from Pt/Cu single atom alloys

Supplementary Figure 1 shows TPD traces of CO from 0.02 ML Pt/Cu(111) surface saturated with CO (10 L). The 4 peaks <250 K correspond to the 3 packing structures of CO on Cu(111) and CO desorption from CO steps.[4–6] After deposition of Pt, an additional peak is seen for CO desorbing from the Pt sites at 350 K. The desorption of CO from isolated Pt sites is ~100 K lower than the desorption observed from Pt(111) (450 K) demonstrating the reduced binding strength of CO to single atom alloys.[7]

Supplementary Note 2: Hydrogenation of butadiene using temperature programmed desorption/reactions

Sub-monolayer (0.2 ML) desorption of butadiene from Cu(111) agrees with previously reported desorption profiles for butadiene (Supplementary Figure 2).[8] Butadiene desorbs from Cu(111) at 220 K and from Cu steps at 330 K. After the deposition of Pt, we observe an additional desorption peak at 290 K (Supplementary Figure 3) which is consistent with butadiene desorption from individual Pt atom sites. Pt atoms do not induce decomposition of butadiene or self-hydrogenation since we do not observe any desorption features for H_2 after the adsorption of butadiene on 0.02 ML Pt/Cu(111). The small amount of butenes desorbing at 230 K arises from a minor impurity in the butadiene sample.

We adsorbed 0.2 ML 1-butene onto 0.02 ML Pt/Cu(111) and butenes desorbed at 240 K (Supplementary Figure 4). Reactively formed butenes and deposited 1-butene desorb at similar temperatures suggesting the overall hydrogenation reaction of butadiene is desorption rate limited.

In order to further investigate the ability of Pt/Cu(111) to hydrogenate butenes, the 0.02 ML Pt/Cu(111) surface was exposed to 50 L H_2 and 0.1 L 1-butene (Supplementary Figure 5). No hydrogenation products were observed.

Supplementary Note 3: Nanoparticle catalyst characterization

As described in Boucher et al.,[1] the Cu NPs were prepared without a support. The average particle size of colloidal Cu NPs is 4.24 ± 1.00 nm with a narrow particle size distribution (Supplementary Figure 6). The Al_2O_3 powder was subsequently introduced to the Cu@PVP colloidal solution to effect Cu@PVP deposition on the support. The alumina- supported Cu@PVP particles were calcined in air to 350 °C to burn off the PVP surfactant. A typical TEM image of the calcined Cu_{15}/Al_2O_3 is shown in Supplementary Figure 6. Supplementary Figure 7 shows the high resolution TEM (HRTEM) images of Cu_{15}/Al_2O_3. The lattice spacing of Cu facets is consistent with that of metallic Cu (Supplementary Figure 7B). Additionally, we found the average crystalline size of Cu NPs increased to 13 nm upon the heat treatments, as determined from the X-ray diffractograms in Supplementary Figure 9 by the Scherrer equation. Boucher et al. reported the sintering of Cu NP during the similar heat treatment in air, and they showed the particle size of Cu NP was more than double as a result of this process.[1]

Complete Pt uptake was verified by UV-Vis spectroscopy of the GR filtrates after replacement of Cu with Pt, as shown in Supplementary Figure 8. In contrast to $PtCl_6^{2-}$ standard, the GR wash solution did not show any absorption band at 260 nm. The broader band in the range of 600 – 800 nm indicates the presence of Cu^{2+} in the GR wash solution.[9] The $PtCl_6^{2-}$ absorption peaks at 260 nm of the standard solutions are consistent with those reported in the literature.[10] ICP-AES was used to quantify Pt in the wash solution and no Pt was detected. The observed concentration of Cu^{2+} is (~ 4 times) more than the Cu^{2+} produced in GR process. The extra Cu^{2+} is a result of some dissolution of surface CuO in acidic solution ($CuO + 2 H^+ \rightarrow Cu^{2+} + H_2O$).[11] The Cu NPs were shortly exposed to the air when transferred from the reduction furnace (100 % H_2, 300 °C) to GR solution. The surface CuO layer that would otherwise impede the GR process was removed by the acid in the solution.

XRD patterns of selected samples are shown in Supplementary Figure 9. Fresh $Pt_{0.2}Cu_{12}/Al_2O_3$ and $Pt_{0.2}Cu_{12}/Al_2O_3$ has been used in the hydrogenation of butadiene have similar XRD patterns as

Cu_{15}/Al_2O_3. Only the metallic Cu phase was detected in $Pt_{0.2}Cu_{12}/Al_2O_3$ and used $Pt_{0.2}Cu_{12}/Al_2O_3$. Any CuO skin formed by exposure of the samples to air upon transfer for analysis was too little to show by XRD. No shift in the Cu-peaks was observed between $Pt_{0.2}Cu_{12}/Al_2O_3$ and used $Pt_{0.2}Cu_{12}/Al_2O_3$ or Cu_{15}/Al_2O_3. This suggests the addition of small amounts of Pt did not change the lattice structure of Cu, which is consistent with the lattice spacing determined by STEM (Figure 5). No CuO or Pt phases were observed by XRD. However, Pt-Cu alloy phases were present in Pt_2Cu_6/Al_2O_3 and the Cu phase diminishes, which indicates the formation of bulk Pt/Cu alloys. Furthermore, no significant change in the XRD spectra was found between fresh and used catalysts. The average crystallite size of Cu_{15}/Al_2O_3 was estimated to be 13 nm based on FWHM of Cu peaks as determined by the Scherrer equation.

Supplementary Figure 10 shows the elemental mapping by energy dispersive X-ray spectroscopy (EDS) of Pt, Cu, Al, and O. The specimen was mounted on a carbon-film-coated Be grid rather than a Cu grid, to preclude any contribution to the EDS signal from the bulk Cu of the grid. By comparing the maps of different elements, we found the Pt was only distributed over the Cu NPs and not on the ϒ-Al_2O_3 support. Moreover, the EXAFS model fitting shows no Pt – O bonds formed in Pt/Cu SAAs (see below, Supplementary Figure 13, Supplementary Table 2). We conclude that the Pt/Cu bimetallic NPs were formed without creating Pt monometallic NPs, which is consistent with the exchange of Pt and Cu atoms in the GR reaction.

The surface composition of Pt/Cu alloys is affected by the gaseous atmosphere, as expected on the basis of the total free energy minimization.[12] Pt is preferentially located at the surface layer of Pt/Cu alloy in H_2 atmospheres, which agrees with the reports that annealing Cu coated Pt(111) in H_2/Ar atmosphere at 400 °C for 2 min led to segregation of Pt that the surface was fully covered by Pt.[13] Since the Pt/Cu catalysts in this work were reduced in 100% H_2 at high temperatures prior to the hydrogenation reaction in a hydrogen-rich gas mixture, we expect the Pt species to be distributed on the surface of the NPs.

To gain insight into the electronic states of the Pt species, XANES spectra were collected for Pt monometallic and Pt/Cu bimetallic samples at Pt-L_{III} edge (Supplementary Figure 11). The Pt/Cu bimetallic and Pt monometallic samples were reduced in 100 % H_2 *in situ* prior to the XANES scans. The white line intensity of Pt/Cu samples is significantly lower than that of Pt^{4+}, Pt^{2+}, Pt foil and Pt NPs indicating that the electronic states of Pt are altered in the Pt/Cu bimetallic NPs. Lee and co-workers have reported the decrease in Pt-L_{III} white line intensity of CuPt alloys compared to pure Pt. The change was attributed to changes in Pt $d_{5/2}$ density of states upon alloying.[14] Since Pt-L_{III} edge characterizes the Pt d states, the decrease in white line intensity of Pt/Cu samples compared to $Pt_{0.1}/Al_2O_3$ and Pt foil indicates the Pt d states were affected in the Pt/Cu alloy samples.[15]

The *in situ* EXAFS spectra of Pt/Cu bimetallic samples and Pt foil are plotted in k- and Fourier transform spaces (Figure 5E and Supplementary Figure 13). EXFAS of $Pt_{0.1}Cu_{14}/Al_2O_3$ and $Pt_{0.2}Cu_{12}/Al_2O_3$ are significantly distinct from that of Pt foil and Pt_2Cu_6/Al_2O_3. Indeed, no Pt-Pt bonds were formed in $Pt_{0.2}Cu_{12}/Al_2O_3$ or $Pt_{0.1}Cu_{14}/Al_2O_3$. Also, no Pt-O bonds were detected in the Pt/Cu NPs samples (Supplementary Table S2). The Pt-Cu coordination number (CN) is 10.45 for $Pt_{0.1}Cu_{14}/Al_2O_3$ and 10.10 for $Pt_{0.2}Cu_{12}/Al_2O_3$, which are between a CN of 12 for the bulk and 9 for the surface inferring that Pt atoms reside in both the surface and bulk. Though galvanic replacement method exchanges surface atoms, the catalyst samples have been pre-reduced in pure H_2 at 350°C prior to the EXAFS measurements. At this temperature the Pt and Cu atoms have significant mobility to diffuse into the bulk of the nanoparticles.[16,17] Due to the error in CN measurements, we cannot conclude the absolute distribution of Pt in Cu host based on EXAFS.

The Pt/Cu SAA NPs in $Pt_{0.1}Cu_{14}/Al_2O_3$ is imaged by the ac-HAADF-STEM after reduction in H_2 at 350 °C. The isolated Pt atoms are brighter than the Cu areas in the dark field STEM images. So we are able to identify the isolated Pt atoms from its higher brightness comparing to its surrounding area. As shown in Supplementary Figure 14, the isolated Pt atoms are dispersed on the Cu NPs. The white circles

highlight some of the isolated Pt atoms. The background from Cu NPs in the ac-HAADF-STEM images is not uniform. Assuming a spherical shape of the NPs, the center region appears brighter due to the greater contribution from the Cu as highlighted by black circles. However, within localized regions, the background remains constant and we observe the bright Pt atoms surrounded by dark Cu regions, which is the evidence for the formation of isolated Pt atoms. Besides that, we do not observe any single atoms or fine clusters of Pt atoms on the alumina.

Supplementary Note 4: Selective hydrogenation of butadiene

The selective hydrogenation activity of the catalysts was studied at near-ambient temperature using lower space velocity, as shown in Figure 6. After reduction in 100 % H_2 at 350 °C for 4 h, a gas mixture of 1.25 % 1,3-butadiene, 20 % H_2 and balance He (total flow rate=20 ml/min, catalyst load=400mg, GHSV=1,200 h^{-1}) was introduced at 120 °C. The temperature was decreased to desired values, and held for 10 min prior to GC injections. The yields of different products are shown in Supplementary Figure 15. Under the conditions employed, the yield to butane over $Pt_{0.1}/Al_2O_3$ and Pt_2Cu_6/Al_2O_3 was close to zero.

The stability of Pt/Cu SAA catalysts in butadiene hydrogenation reaction is demonstrated at 160 °C. As shown in Supplementary Figure 16, the production rates of butene isomers (the desired products) and the selectivity to butenes are stable for at least 46 hours. Please note the space velocity here is greater (12,000 h^{-1} versus 1,200 h^{-1}) than that of Figure 6 and Supplementary Figure 15. The spent catalyst used for the stability test was tested in He-TPD and TPO whereby no desorption of hydrocarbons took place and a negligible amount of CO_2 was produced (see below, Supplementary Figure 19), respectively.

Under the same reaction conditions, the Pt/Cu SAAs samples were tested in consecutive ascending and descending temperature cycles. Supplementary Figure 17 shows the conversion of butadiene in a cycle over $Pt_{0.1}Cu_{14}/Al_2O_3$. The comparison of conversion at 150 °C and 120 °C between the increasing and decreasing temperature ramps shows that the hydrogenation activity was recovered during the decreasing temperature ramp. But at near ambient temperatures, the activity in the cooling part of the cycle is lower. The same was true with all of the Pt/Cu alloyed samples. The results suggest the hydrogenation activity was reduced at lower temperatures but recovered at higher temperatures. Considering that oligomer formation adversely affects the metal-based catalysts in the hydrogenation of hydrocarbons at mild temperatures,[18] we hypothesize the oligomers partially blocked the surface sites at lower temperature and desorbed with increasing temperature.

To check this, after the first temperature heating-cooling cycle (Supplementary Figure 17A), the catalyst was treated at 350 °C in H_2 to fully desorb the weakly adsorbed hydrocarbons on the surface. We found the selective hydrogenation activity was fully recovered after the high temperature treatment for at least 4 cycles, as shown in Supplementary Figure 17B. This result suggests the active sites were maintained in the cyclic reactions.

To demonstrate the superior catalytic activity of Pt/Cu SAAs, selective hydrogenation of butadiene was performed over the Pt and Cu monometallic samples under the same conditions (GHSV=12,000 h^{-1}, 0.1 g catalyst, flow rate = 50 ml/min. 2 % 1, 3-butadiene, 20 % H_2 and balanced He). Supplementary Figure 18 shows the hydrogenation rates measured on different catalysts at 30, 60, 90 and 120 °C. The concentration of different products were measured with a GC-FID and the overall production rates were calculated. The production rates were calculated by dividing the overall production rate by the total weight of the catalysts. The Pt/Cu SAA catalysts ($Pt_{0.1}Cu_{14}/Al_2O_3$ and $Pt_{0.2}Cu_{12}/Al_2O_3$) show the best performance in terms of the yield to butenes, the desired products. The enhancements in selective hydrogenation activity of Pt/Cu SAAs compared to Cu monometallic samples

is due to the Pt atoms activating the H_2 at near ambient temperature, as shown in the single crystal surface studies. While the Pt monometallic catalyst is active in the hydrogenation of butadiene, it is much less selective compared to Cu monometallic catalysts or Pt/Cu SAAs, which leads to lower yields to butenes. Pt/Cu SAA catalysts show superior activity and selectivity compared to their monometallic counterparts, making them promising candidates for use in industrial selective hydrogenation processes.

The selectivity of Pt/Cu SAAs in excess propylene demonstrates the viability of SAAs use in industrial alkene feedstocks.[19] Below 120 °C, propylene hydrogenation was not observed with Pt/Cu SAAs (Supplementary Figure 19). Long time selective hydrogenation tests were performed at 160 °C and 145 °C, at which the conversion was nearly 100 %. We found the high selectivity in hydrogenation of butadiene was maintained for at least 12 h (Figure 7).

TPO and He-TPD were performed on both of the spent catalysts that have been used in 48 h hydrogenation of butadiene (Supplementary Figure 20B) and 30 h hydrogenation in the presence of propylene (Supplementary Figure 20A). In TPO test, CO_2 produced by the combustion of carbon species on the catalyst was monitored by mass spectrometry (m/z=44), as shown in Supplementary Figure 20. We found that carbon deposition on the catalyst was negligible. The CO_2 concentration was below 0.02 % with TPO performed up to 800 °C. Moreover, for the catalysts used in the hydrogenation of butadiene (Supplementary Figure 20B), the total CO_2 production corresponds to less than 1/26,000 of the amount of butadiene converted over the catalysts. For another spent catalyst (Supplementary Figure 20A), the CO_2 production in TPO corresponds to less than 1/90,000 of the amount of hydrocarbon flowed through the catalysts during the hydrogenation of butadiene in the presence of propylene. Similarly, He-TPD shows no desorption of hydrocarbons up to 800 °C as indicated by the lack of hydrocarbon fragments in the range of m/z=1-120 for both spent catalysts.

Supplementary Methods

For the preparation of nanoparticles, metal precursors chloroplatinic acid hydrate ($H_2PtCl_6 \cdot x H_2O$, ~38% Pt basis, Sigma-Aldrich), and copper nitrate trihydrate ($Cu(NO_3)_2 \cdot 3 H_2O$, Johnson Matthey) were used. Aluminum oxide (ϒ-Al_2O_3, ultra-pure grade 99.99%, surface area 70-100 m^2/g, Inframat Advanced Materials) was used as catalyst support. Poly(vinylpyrrolidone) (PVP, MW= 58,000, Alfa Aesar) was used as stabilizing agent for colloidal nanoparticles. L-ascorbic acid (Sigma-Aldrich) was used as anti-oxidizing agent. The copper precursor was reduced with sodium borohydride ($NaBH_4$, Sigma-Aldrich) to form the nanoparticles (NP).

Pt/Cu bimetallic samples were synthesized by galvanic replacement (GR) reaction as described below. The method of preparing Cu NPs was reported elsewhere.[1] Briefly, an aqueous solution of $Cu(NO_3)_2$ and PVP (200:1 molar ratio of Cu to PVP, 1:1 wt/wt) was degassed in vacuum and in nitrogen gas sequentially. A 0.1 M solution of ascorbic acid was added to the Cu/PVP solution, followed by the addition of $NaBH_4$ solution (0.1 M) dropwise. The solution turned to an opaque brown suspension after the addition of $NaBH_4$. Aluminum oxide (2 g) was suspended in 100 ml of DI water under magnetic stirring and added to the Cu NPs colloidal solution dropwise. The solution was kept under flowing nitrogen and constant stirring for 30 min before it was filtered and washed with DI water 3 times. The resulting materials were dried in vacuum for 24 h, calcined in air to 350 ^{o}C at 2 ^{o}C/min and held for 4 h.

GR took place in aqueous solution under nitrogen protection with constant stirring and refluxing at 100 °C. The desired amount of Pt precursor ($H_2PtCl_6 \cdot x H_2O$, Sigma - Aldrich) was added to the suspension of Cu NPs (pre-reduced in 100% H_2 at 300 °C) containing HCl (2mM). The Pt precursor was reduced by Cu host via the displacement reaction shown in Scheme 1. After 20 min, the resulting material was filtered, washed and dried in vacuum. The sample composition is listed in Table S1.

$PtCl_6^{2-} + 4 e^- \rightarrow Pt + 6 Cl^-$ ($\Delta E° = 0.74$ V)
$Cu \rightarrow Cu^{2+} + 2 e^-$ ($\Delta E° = -0.34$ V)

Scheme 1. GR reaction.

HRTEM was conducted on a JEOL 2010 electron microscope with 200 kV and 107 μA beam emission. The specimens were obtained by drying one drop of colloidal solution of NPs in ethanol onto a carbon film with nickel or copper covered microgrid.

Ultraviolet and visible light (UV-Vis) absorption spectra were collected in the range of 200 - 800 nm using an Evolution™ 300 UV-Vis Spectrophotometer (Thermo Scientific).

X-ray photoelectron spectra (XPS) were obtained by using a Thermo Scientific K-Alpha system equipped with an Al source and a double focusing hemispherical analyzer with a 128-channel detector at a pass energy of 50 eV.

X-ray powder diffraction (XRD) analysis was performed on a PANalytical X'Pert Pro instrument. Cu Kα radiation was used with a power setting of 40 mA, 45 kV. Data was collected for 2θ between 20° and 80°. The catalysts were reduced in 100% H_2 at 350 °C (Pt/Cu samples) or 300 °C (Cu monometallic samples) prior to the XRD measurements.

The Pt and Cu loading on each sample was measured by inductively coupled plasma ion emission spectroscopy (ICP/IES) on a Leeman Labs PS1000 instrument. The solid samples were digested in concentrated HCl and H_2O_2 prior to the testing. No Pt or Cu was detected by XPS on the solid alumina support after digestion. The collected wash solution from GR and Cu NP preparation was used without additional treatments.

X-ray absorption spectroscopy (XAS) was performed at beamline X18B at Brookhaven National Laboratory and beamline 12-BM at Argonne National Laboratory. The Pt/Cu and Pt NPs were reduced in 100% H_2 at 350 °C (Pt/Cu bimetallic samples) or 400 °C (Pt monometallic samples) *in situ* prior to the tests. The X-ray absorption near edge structure (XANES) spectra were collected in H_2 or He atmospheres.

Extended X-ray absorption fine structure (EXAFS) was measured in H_2. The spectra were recorded in the fluorescence mode at room temperature.

XAS data were processed and analyzed using Athena and Artemis.[2] The XANES spectra were background corrected and normalized. And the EXAFS data were fitted in r-space with the models based on metallic Pt, $PtCu_3$, and PtO.[3]

The hydrogenation activity of the catalysts was tested in a packed-bed flow microreactor (L=22 inch, O.D.=1/2 inch) with 100 mg of catalyst diluted by 0.5 g of quartz particles. The samples were pre-reduced in 100% H_2 at 25 ml/min (300 °C for Cu_{15}/Al_2O_3, 350 °C for Pt/Cu samples and 400 °C for $Pt_{0.1}/Al_2O_3$) with a heating rate of 2 °C/min, and then cooled to 30 °C before the reaction gas mixture (2 % 1,3-butadiene, 20 % H_2 and balance He. GHSV=12,000 h^{-1}) was introduced. The tests were started at 30 °C and the temperature was raised to higher values (60, 90, 120, 150 and 170 °C) at 5 °C/min and held at each temperature for 30 min. After reaching 170 °C, the samples were cooled back to RT, collecting data at selected intermediate temperatures to check for (de)activation. Gas chromatograph (GC) injections were done by sampling the gas at various temperatures after stabilizing the temperature for 10 min. Moreover, long-time stability tests were conducted isothermally at 160 °C for 46 h following the activity tests (2 % 1,3-butadiene, 20 % H_2 and balance He. GHSV=12,000 h^{-1}). The exit gas stream was analyzed by a HP6890 GC equipped with a flame ionization detector (FID). A 30 ft. column (1/8 in. dia.) filled with Sebaconitrile 20 % Chromosorb Paw 80/100 supplied by Sigma-Aldrich was used to separate the gas species.

The activity tests with added propylene were conducted in a similar fashion with a gas mixture of 2 % 1,3-butadiene, 20 % propylene, 16 % H_2 and balance He. The total flow rate was 50 ml/min, and the catalyst load was 100mg (GHSV=12,000 h^{-1}). The steady-state stability tests were conducted isothermally at 160 °C and 145 °C, for 12 h at each temperature. The exit gas stream composition was determined by GC as described above.

Cyclic hydrogenation tests were performed to probe the stability of the catalysts. The first cycle was performed as described for the activity test. Then the sample was treated in 100 % H_2 at 350 °C for 1 h. The reaction gas mixture (2 % 1,3-butadiene, 20 % H_2 and balance He. GHSV=12,000 h^{-1}) was introduced at 170 °C followed by cooling down to other temperatures. GC injections were done at specified temperatures after the temperature was held stable for at least 10 min.

Temperature- programmed oxidation (TPO) was conducted in a Micromeritics AutoChem II 2920 instrument equipped with a mass spectrometer. The spent catalyst (33 mg) used in the long-time stability testing with or without addition of propylene was loaded to the microreactor for the TPO test. The sample was degassed with He at 10 °C/min up to 100 °C, and cooled to 25 °C in He. Next, 20 % O_2 in He was introduced. The sample was stabilized at 25 °C for 5 min followed by raising the temperature to 800 °C at 3 °C/min. The CO_2 in the gas stream was analyzed online by a mass spectrometer (Pfeiffer, TCP270). Temperature programmed desorption in He (He-TPD) was performed with a similar setup. The spent catalyst (33 mg) from the long-time stability testing with or without addition of propylene was loaded to the micro reactor for the He-TPD test. After degassing in pure He flow (50 ml/min) for 20 min at ambient temperature, the sample was heated in the He flow (50 ml/min) to 800 °C at 3 °C/min. The exit gas stream was analyzed online by a mass spectrometer in histogram-spectrum mode scanning mass-to-charge ratio (m/z) between 1 and 120.

Supplementary References

1. Boucher, M. B. *et al.* Single atom alloy surface analogs in $Pd_{0.18}Cu_{15}$ nanoparticles for selective hydrogenation reactions. *Phys. Chem. Chem. Phys.* **15,** 12187–12196 (2013).
2. Ravel, B. & Newville, M. ATHENA, ARTEMIS, HEPHAESTUS: Data analysis for X-ray absorption spectroscopy using IFEFFIT. in *J. Synchrotron Radiat.* **12,** 537–541 (2005).
3. Ravel, B. ATOMS: Crystallography for the X-ray absorption spectroscopist. *J. Synchrotron Radiat.* **8,** 314–316 (2001).
4. Raval, R. *et al.* FT-Rairs EELS LEED Studies of the adsorption of carbon monoxide on Cu(111). *Surf. Sci.* **203,** 353–377 (1988).
5. Kirstein, W., Krüger, B. & Thieme, F. CO adsorption studies on pure and Ni-covered Cu(111) surfaces. *Surf. Sci.* **176,** 505–529 (1986).
6. Kneitz, S., Gemeinhardt, J. & Steinru, H. A molecular beam study of the adsorption dynamics of CO on Ru (0001), Cu (111) and a pseudomorphic Cu monolayer on Ru (0001). *Surf. Sci.* **440,** 307–320 (1999).
7. Collins, D. M. & Spicer, W. E. The adsorption of CO, O_2 and H_2 on Pt. *Surf. Sci.* **69,** 85–113 (1977).
8. Huang, W., Wei, W., Zhao, W. & White, J. M. Two-photon photoemission spectroscopy study of 1 , 3-butadiene on Cu (111): electronic structures and excitation mechanism. *J. Phys. Chem. B* **110,** 5547–5552 (2006).
9. Sun, Z. *et al.* Rapid and surfactant-free synthesis of bimetallic Pt-Cu nanoparticles simply via ultrasound-assisted redox replacement. *ACS Catal.* **2,** 1647–1653 (2012).
10. Hikosaka, K., Kim, J., Kajita, M., Kanayama, A. & Miyamoto, Y. Platinum nanoparticles have an activity similar to mitochondrial NADH:ubiquinone oxidoreductase. *Colloids Surf. B. Biointerfaces* **66,** 195–200 (2008).
11. Iwai, M., Majima, H. & Awakura, Y. Dissolution of Copper in hydrochloric acid solutions with dissolved moleculaoxygen. *Hydrometallurgy* **20,** 87–95 (1988).
12. Van Langeveld, A. D. & Ponec, V. The surface composition of Pt-Cu Alloys; Experimental observations and theory of surface segregation. *Appl. Surf. Sci.* **16,** 405–423 (1983).
13. Calle-Vallejo, F., Koper, M. T. M. & Bandarenka, A. S. Tailoring the catalytic activity of electrodes with monolayer amounts of foreign metals. *Chem. Soc. Rev.* **42,** 5210–30 (2013).
14. Lee, Y., Lim, K., Chung, Y., Whang, C. & Jeon, Y. XPS core-level shifts and XANES studies of Cu – Pt and Co – Pt alloys. **478,** 475–478 (2000).
15. Mansour, A. N., Cook, J. W. & Sayers, D. E. Quantitative rechnique for the determination of the number of unoccupied d-electron states in a platinum catalyst using the $L_{2,3}$ X-ray absorption edge spectra. *J. Phys. Chem.* **88,** 2330–2334 (1984).
16. Martin, A. B., Johnson, R. D. & Asaro, F. Diffusion of gold into copper. *J. Appl. Phys.* **25,** 364–369 (1954).
17. Butrymowicz, D. B., Manning, J. R. & Read, M. E. Diffusion in copper and copper alloys, Part II. Copper-silver and copper-gold Systems. *J. Phys. Chem. Ref. Data* **3,** 527 (1974).
18. Delannoy, L. *et al.* Selective hydrogenation of butadiene over TiO_2 supported copper, gold and gold-copper catalysts prepared by deposition-precipitation. *Phys. Chem. Chem. Phys.* **16,** 26514–27 (2014).
19. Derrien, M. L. Selective hydrogenation applied to the refinging of petrochemical raw materials produced by steam cracking. *Stud. Surf. Sci. Catal.* **27,** 613–666 (1986).
